# Genome-Wide Association Studies Reveal Genomic Regions Associated With the Response of Wheat (*Triticum aestivum* L.) to Mycorrhizae Under Drought Stress Conditions

**DOI:** 10.3389/fpls.2018.01728

**Published:** 2018-12-04

**Authors:** Heike Lehnert, Albrecht Serfling, Wolfgang Friedt, Frank Ordon

**Affiliations:** ^1^Institute of Federal Research Centre for Cultivated Plants, Institute for Resistance Research and Stress Tolerance, Julius Kühn-Institute (JKI), Quedlinburg, Germany; ^2^IFZ Research Centre for Biosystems, Land Use and Nutrition, Plant Breeding Department, Justus Liebig University, Gießen, Germany

**Keywords:** 90k iSelect chip, arbuscular mycorrhizae, bread wheat reference genome, drought stress tolerance, genome-wide association study (GWAS), mycorrhizal responsiveness, quantitative trait loci (QTLs), *Triticum aestivum* L. (bread wheat)

## Abstract

In the majority of wheat growing areas worldwide, the incidence of drought stress has increased significantly resulting in a negative impact on plant development and grain yield. Arbuscular mycorrhizal symbiosis is known to improve drought stress tolerance of wheat. However, quantitative trait loci (QTL) involved in the response to drought stress conditions in the presence of mycorrhizae are largely unknown. Therefore, a diverse set consisting of 94 bread wheat genotypes was phenotyped under drought stress and well watered conditions in the presence and absence of mycorrhizae. Grain yield and yield components, drought stress related traits as well as response to mycorrhizae were assessed. In parallel, wheat accessions were genotyped by using the 90k iSelect chip, resulting in a set of 15511 polymorphic and mapped SNP markers, which were used for genome-wide association studies (GWAS). In general, drought stress tolerance of wheat was significantly increased in the presence of mycorrhizae compared to drought stress tolerance in the absence of mycorrhizae. However, genotypes differed in their response to mycorrhizae under drought stress conditions. Several QTL regions on different chromosomes were detected associated with grain yield and yield components under drought stress conditions. Furthermore, two genome regions on chromosomes 3D and 7D were found to be significantly associated with the response to mycorrhizae under drought stress conditions. Overall, the results reveal that inoculation of wheat with mycorrhizal fungi significantly improves drought stress tolerance and that QTL regions associated with the response to mycorrhizae under drought stress conditions exist in wheat. Further research is necessary to validate detected QTL regions. However, this study may be the starting point for the identification of candidate genes associated with drought stress tolerance and response to mycorrhizae under drought stress conditions. Maybe in future, these initial results will help to contribute to use mycorrhizal fungi effectively in agriculture and combine new approaches i.e., use of genotypic variation in response to mycorrhizae under drought stress conditions with existing drought tolerance breeding programs to develop new drought stress tolerant genotypes.

## Introduction

Wheat (*Triticum aestivum* L.) is one of the most important staple food crops worldwide with an annual production of 749 million tons in 2016 (Faostat, 2018). The demand for wheat is increasing constantly, as human population is growing continually (Godfray et al., 2010; Tilman et al., 2011). Since the green revolution in the 1960s, wheat yield has increased steadily, mainly by improving the harvest index. However, during the last two decades the increase in yield per hectare is stagnating in Europe (Brisson et al., 2010; Ahlemeyer and Friedt, 2011; Rybka and Nita, 2015). It is assumed that this is due to changes in weather conditions and the negative effects of increasing number of abiotic stress events, like heat and early summer drought (Brisson et al., 2010; Ahlemeyer and Friedt, 2011). In fact, since 1980, in the majority of wheat growing areas worldwide, trends in temperature, and precipitation have changed significantly combined with an increased incidence of drought stress events (Lobell et al., 2011; Coumou and Rahmstorf, 2012; Zampieri et al., 2017) having negative effects on plant development and yield due to e.g., premature leaf senescence, decreased leaf water potential, stomatal closure, reduced net photosynthesis, oxidative damage of chloroplasts and reduced rates of carbon fixation and assimilate translocation (reviewed in Farooq et al., 2014; reviewed in Osakabe et al., 2014; reviewed in Rao and Chaitanya, 2016). At any stage of plant development, drought stress affects plant growth, whereby terminal drought stress during flowering and grain filling is related to maximum yield losses (reviewed in Farooq et al., 2014). Plants developed several mechanisms to maintain growth under drought stress conditions i.e., a robust root system, leaf area reduction, deposition of epicuticular waxes, expression of stress responsive genes, hormonal regulation, accumulation of osmolytes, modulation of the antioxidant system, delayed senescence, or reverse translocation of assimilates (reviewed in Farooq et al., 2014; reviewed in Kulkarni et al., 2017; reviewed in Wang and Qin, 2017). These numerous responses to drought require genome-wide reprogramming of gene expression and metabolism (Ma et al., 2017). Additionally, it has also been shown that arbuscular mycorrhizal symbiosis improves drought stress tolerance of plants (Jayne and Quigley, 2014). Furthermore, recent research suggests that inoculation of plants with these beneficial soil fungi also affects modulation of proteins related to drought stress response and therefore reduces osmotic stress (Bernardo et al., 2017).

Arbuscular mycorrhizal symbioses, i.e., the symbiotic association between plants and obligate and biotrophic fungi of the phylum Glomeromycota, are the oldest types of plant microbe interactions (Brundrett, 2002; Schüßler and Walker, 2010). These associations are as old as the land plants themselves, and have evolved even before roots around 400 million years ago (Taylor et al., 1995) thereby significantly contributing to the terrestrialization of plants by providing water and nutrients (Humphreys et al., 2010). Today, more than 80% of land plants including cultivated species e.g., bread wheat, rice (*Oryza sativa*) and maize (*Zea mays*) form mycorrhizal symbioses, which are characterized by the exchange of photosynthetic products (predominantly glucose) produced by the plant and nutrients and water supplied by the fungus across the symbiotic interface (Solaiman and Saito, 1997; Smith and Read, 2008; An et al., 2010; Nakagawa and Imaizumi-Anraku, 2015; Pellegrino et al., 2015). Therefore, in general, this type of interaction is considered to be mutualistic (Smith and Read, 2008). Positive effects on plant performance by interactions with these soil microbes are most prominent under growth limiting conditions e.g., drought, salinity, or poor nutrient availability due to a better adaption or improved water and nutrient supply of mycorrhizae colonized plants (Sharif et al., 2011; Augé et al., 2014; Jayne and Quigley, 2014). However, a continuum of plant responses to mycorrhizae exist depending on environmental conditions, plant species and genotype, and the interaction between genotype and mycorrhizae species (Johnson et al., 1997, 2015). Therefore, interaction between both partners can be described as mutualism, commensalism, or parasitism (Johnson et al., 1997). The most common method to record the response of plants to mycorrhizae is the calculation of the mycorrhizal responsiveness (Hetrick et al., 1992; Janos, 2007; Sawers et al., 2010). For several plant species including wheat, it has been shown that genotypic differences in response to mycorrhizae exist (Hetrick et al., 1992; Kaeppler et al., 2000; Yücel et al., 2009; Galván et al., 2011).

It is to be expected that drought stress events will occur more frequently in the future due to the increase of heat extremes (Coumou and Robinson, 2013). Therefore, in the future, the availability of high yielding genotypes better adapted to drought stress will become of prime importance (Gupta et al., 2017). Until now, the most promising approach to increase drought stress tolerance of wheat is methodical breeding (Farooq et al., 2014). In this respect, drought stress tolerance of plants is in general assessed on the basis of agronomic performance as well as physiological and root related traits under drought stress conditions (Gupta et al., 2017). These drought stress-associated traits are complex and polygenic in nature and, therefore conventional breeding of drought stress tolerant genotypes is difficult and progress is slow (Edae et al., 2014; Gupta et al., 2017; Wang and Qin, 2017). In addition, today, it is proposed to use the genotypic differences of plants in response to mycorrhizae in breeding programs to improve both the ability to respond to mycorrhizae and the plant performance *per se* (Fester and Sawers, 2011; Galván et al., 2011). However, until now, in plant breeding, no practical application of genotypic differences in plant response to mycorrhizae has been reported (Galván et al., 2011).

Today, genotypic differences can be used effectively in plant breeding by identifying quantitative trait loci (QTL) associated with the trait of interest via genome-wide association studies (GWAS) or bi-parental QTL mapping studies followed by marker assisted selection (Rafalski, 2010). GWAS based on linkage disequilibrium (LD) to identify QTL associated with the trait of interest is nowadays widely applied in plant genetics and breeding (Ingvarsson and Street, 2011; Lipka et al., 2015). In wheat, several QTL regions associated with complex traits were identified by GWAS (e.g., Zhang et al., 2014; Zanke et al., 2015; Hoffstetter et al., 2016; Sun et al., 2017). However, only a few reports are known on the identification of QTL regions associated with drought stress related traits in wheat by GWAS (Zhang et al., 2013; Edae et al., 2014; Ain et al., 2015; Lopes et al., 2015; Mwadzingeni et al., 2017). The majority of drought stress associated QTL regions was detected by QTL mapping studies (reviewed in Acuña-Galindo et al., 2015; reviewed in Gupta et al., 2017). Furthermore, QTL associated with response to mycorrhizae have been identified for maize and *Allium* spp (Kaeppler et al., 2000; Galván et al., 2011). Until now, little is known about the genetic basis of wheat in response to mycorrhizae (Hetrick et al., 1995; Yücel et al., 2009) and no information is available about QTL regions associated with response to mycorrhizae under drought stress conditions.

Identification of QTL regions associated with drought stress tolerance *per se* or the response to mycorrhizae under drought stress conditions is the first step toward the identification of candidate genes as well as the underlying molecular mechanisms and the development of gene-based functional markers suited for marker based selection to enhance the improvement of drought stress tolerance in wheat (Gupta et al., 2017).

Therefore, the objectives of this study were (i) to obtain information on genetic differences with regard to drought stress tolerance of wheat and the response of wheat to mycorrhizae under drought stress conditions, and to (ii) identify QTL regions involved in drought stress tolerance and response to mycorrhizae by GWAS followed by (iii) the identification of candidate genes located in the QTL regions.

## Materials and Methods

### Plant Material, Inoculum and Experimental Design

A set of 94 winter wheat cultivars consisting of German wheat cultivars (registered between 1950 and 2010 or before 1945) and a worldwide wheat collection derived from 21 different countries was evaluated for drought stress tolerance in the presence and absence of mycorrhizae (Supplementary Table 1)

Three mycorrhizae species, i.e., *Rhizophagus intraradices, Claroideoglomus claroideum*, and *Claroideoglomus etunicatum*, previously *Glomus intraradices, Glomus claroideum*, and *Glomus etunicatum* (Schüßler and Walker, 2010) were used in this study. All of these mycorrhizae species are known to be able to generate symbiosis with wheat [Hetrick et al., 1992 (*C. etunicatum, R. intraradices*); Bryla and Duniway, 1998 (*C. etunicatum*); Zhu and Smith, 2001 (*R. intraradices*); Zhu et al., 2001 (*R. intraradices*); Beltrano and Ronco, 2008 (*C. claroideum*); Moucheshi et al., 2012 (*C. etunicatum, R. intraradices*)] To produce mycorrhizal and non-mycorrhizal substrates, mycorrhizal sand inoculum (Sprint, INOQ GmbH, Schnaga, Germany; mycorrhiza units per cm^3^ inoculum: 220) or autoclaved sterile sand inoculum (control) was evenly mixed with nutrient poor (average pH: 6, N: 19 mg l^−1^, P_2_O_2_: <4 mg l^−1^, K_2_O: 20 mg l^−1^) peat soil (Archut Fruhstorfer Erde Typ Null, HAWATI Group GmbH, Vechta, Germany) in the ratio 4:96 (w/w), respectively.

Pot trials to examine the drought stress tolerance of wheat in the presence and absence of mycorrhizae were carried out in a glasshouse in 2013 and 2014 at the Julius Kühn-Institute, Quedlinburg (Germany). Experiments were laid out in a split-split plot design [main factor: mycorrhizae (two levels: mycorrhizal vs. non-mycorrhizal treatment); subplot factor: irrigation regime (two levels: well watered vs. drought stress treatment); sub-subplot factor: genotype (1–94)] in three replicates. This type of trial design was chosen to prevent mycorrhizae contamination in the non-mycorrhizal treatment and to maintain drought stress condition in the drought stress variant.

Seven days old seedlings were vernalized (8 weeks at 4°C) in plastic trays containing sterile soil (Archut Fruhstorfer Erde Typ Aussaat- und Stecklingserde, HAWATI Group GmbH, Vechta, Germany). After vernalization, seedlings were transferred to pots (20 × 25.5 cm, 4 seedlings per pot) filled with 2.2 kg of mycorrhizal or non-mycorrhizal substrate, respectively. Basic fertilization was conducted according to Zhu and Smith (2001). Nutrients were diluted in distilled water and per 1 kg substrate the following quantities were added: 0.174g K_2_SO_4_, 0.185g ${\text{MgSO}}_{4}^{*}$7H_2_O, 0.004g FeEDTA, 1.400mg ${\text{CuSO}}_{4}^{*}$H_2_O, 0.460mg ${\text{MnSO}}_{4}^{*}$H_2_O, ${\text{CoSO}}_{4}^{*}$7H_2_O, 0.500mg H_3_BO_3_, 0.400mg MoO_3_, 2.200mg ${\text{ZnSO}}_{4}^{*}$7H_2_O. N-fertilization was conducted at planting (BBCH: 16, 0.918g Ca (NO3)2^*^H2O per 1kg substrate, Zadoks et al., 1974; Hack et al., 1992), at the end of tillering (BBCH: 26-29, 0.530g Ca (NO_3_${)}_{2}^{*}$H_2_O per 1kg substrate), and at booting (BBCH: 41.-43, 0.126g Ca (NO_3_${)}_{2}^{*}$H_2_O per 1kg substrate). Plants were grown under semi-controlled greenhouse conditions from February to June 2013 and 2014, respectively. Periods of light and dark, temperature as well as additional lighting intensity were individually adapted to plant development [planting to early tillering stage: 14 h of light (12 to 15°C) and 10 h of dark (8 to 11°C), tillering to late booting stage: 16 h of light (16 to 18°C) and 8 h of dark (12 to 14°C), as from ear emergence: 16 h of light (20 to 24°C) and 8 h of dark (16 to 18°C)]. Additional lighting was only applied in the absence of sufficient natural sunlight (below 40K lux) during the period of light.

Every 2 days watering was performed by weighting of pots (i.e., water loss). At the early booting stage (BBCH 41 to 43) irrigation treatments were started. From this point until full maturity, maximal soil water capacity (MWC) was maintained at 25 or 75% in the drought stressed and in the well watered variant, respectively.

### Determination of Yield and Yield Components

At full maturity, number of ears per plant (EN) was counted on each plant per pot and aboveground biomass and roots of each pot were harvested. Roots were cleaned of soil and washed several times in tap water. A randomly chosen sample of roots per pot was stored in a solution of 70% ethanol and 99% glacial acetic acid (1:1 ratio; v/v) for further analysis. The remaining roots per pot were dried (105°C for 24 h) and root dry mass per pot (RM; in gram) was determined by weighting. Furthermore, ears were separated from straw and ears (30°C for 8 h) and straw (105°C for 24 h) were dried separately. Dried ears were manually threshed and grain yield per pot was determined in gram by weighting. Mean grain yield per plant (GY; in gram) was calculated as *grain yield per pot*/*number of plants per pot*. Thousand grain weight (TGW; in gram) was calculated by counting and weighting three times 100 grains per pot and multiplying with 10. Number of grains per ear (GN) was calculated as [*grain yield per pot*/(*TGW*/1000)]/*ears per plant*. Straw yield was measured as aboveground biomass without grains (straw; in gram). Mean straw yield per plant (SY) was calculated as *straw yield per pot*/*number of plants per pot*. Mean aboveground biomass yield per plant (BM) was calculated by adding up GY and SY. Harvest index (HI) was calculated as *mean grain yield per plant*/*mean aboveground biomass yield per plant*.

### Determination of Root Colonization

According to the protocol of Vierheilig et al. (1998), a randomly chosen sample of roots per pot was cleared with 10% KOH (w/v), acidified with 3% HCl (v/v) and stained with 5% ink vinegar solution (v/v). For microscopical analyses, 30 evenly stained root pieces (1 to 2 cm) were randomly selected. The magnified intersect method of Mcgonigle et al. (1990) was used for microscopical quantification of root colonization by mycorrhizal fungi. Percentage of root length colonized was assessed.

### Determination of Drought Stress Susceptibility Indices and Response to Mycorrhizae

To estimate the effect of drought stress conditions on yield and yield components of each genotype, two drought stress susceptibility indices (DSI) were calculated, i.e., tolerance index (Kuol, 2004; IT) and stress tolerance index (Fernandez, 1992; STI):

$$\begin{array}{l} \text{ IT}={\left({\text{Y}}_{\text{S}}{\text{/Y}}_{\text{C}}\right)}^{*}100,\\ \text{STI}=\left({\text{Y}}_{\text{S}}^{*}{\text{Y}}_{\text{C}}\right)\text{/}{\left({\text{Y}}_{\text{Call}}\right)}^{2}, \end{array}$$

where Y_S_ = is the respective genotype mean under drought stress conditions, Y_C_ = the respective genotype mean under control conditions and Y_Call_ is the mean of all genotypes under well watered conditions. Drought stress susceptibility indices were calculated separately for each of the mycorrhizae treatments, to evaluate the effect of mycorrhizae on drought stress tolerance of wheat. High values of IT and STI point to better drought stress tolerant genotypes.

To evaluate the changes in yield and yield components associated with mycorrhizae, relative mycorrhizal responsiveness (Hetrick et al., 1992; MR) and absolute mycorrhizal responsiveness (Janos, 2007; Sawers et al., 2010; R) were calculated for each genotype according to the following equations:

$$\begin{array}{l} \text{MR}=\left[\left({\text{Y}}_{\text{m}}-Y_{\text{n}}\right)/Y_{\text{n}}\right]x100,\\ \text{R}=\text{Ym}-\text{Yn}, \end{array}$$

where Y_m_ is the genotype mean of the mycorrhizae treated plants and Y_n_ is the genotype mean of the non-treated plants. MR and R were calculated separately for each of the irrigation regimes, to evaluate the effect of water availability on MR and R of wheat. Negative values of MR and R are indicative for genotypes responding negatively to mycorrhizae, whereas positive values of MR and R are indicative for genotypes responding positively to mycorrhizae.

Additionally, linear regression analysis of performance of genotypes under drought stress conditions in the presence of mycorrhizae against performance of genotypes under drought stress conditions in the absence of mycorrhizae was conducted, by using the software package R (Sawers et al., 2010; R Core Team, 2014). Deviations (δ) from the regression analysis of performance of genotypes under drought stress conditions in the presence of mycorrhizae against performance of genotypes under drought stress conditions in the absence of mycorrhizae were used to evaluate the specific effect of mycorrhizae on genotype specific performance (Sawers et al., 2010). Genotypes showing a positive deviation from the regression line are associated with a high level of specific variation in mycorrhizal responsiveness. Hierarchical cluster analysis (method: Ward) was conducted by using the software package JMP genomics (Jmp® Genomics, 2015).

### Statistical Analyses

Statistical analyses of phenotypic data were performed with the statistics package SAS 9.3 (Sas Institute, 2015). For each of the traits, the procedure PROC MIXED was used for analysis of variance (ANOVA), estimation of least square means (lsmeans) and calculation of differences between lsmeans for all factor combinations (irrigation regime x mycoorrhizae treatment). The effect of genotype was included as a fixed factor in the model to estimate lsmeans for each genotype. A second and a third mixed model was fitted to estimate variance components of each of the factor combinations (irrigation regime x myccorhizae treatment) separately or to estimate variance components of all factor combinations together, respectively. In both models, all effects were considered as random factors. Broad sense heritability (h^2^) and standard error of h^2^ were calculated from variance components using the SAS macro developed by Holland et al. (2003). As root drymass in the non-mycorrhizal variant was only recorded in 2013, repeatability of this trait was calculated instead of h^2^. Phenotypic correlations were estimated with the procedure PROC CORR.

### Genome Wide Association Studies

For each of the 94 genotypes, genomic DNA was extracted from the same plant used for seed production for glasshouse trials, according to the protocol of Stein et al. (2001). Genotyping was conducted at Trait Genetics, Gatersleben (Germany), by using the 90K iSelect chip (Illumina Inc., San Diego, USA), resulting in a raw single nucleotide polymorphism (SNP) marker dataset. The bread wheat reference genome and the genome annotation were downloaded from URGI-INRA (IWGSC, 2018). Next, flanking sequences of raw marker data (Wang et al., 2014) were mapped against the bread wheat reference genome (IWGSC, 2018). All mapped markers were filtered for minor allele frequencies (MAF) >5% and missing values <10%, resulting in a set of 15511 polymorphic, mapped and high quality SNP markers. The filtered data set was imputed for missing values <10% by using the software package Beagle version 3.2.2 (Browning and Browning, 2007) and was used for GWAS.

LD was estimated as squared allele frequency correlation (r^2^) between all pairs of interchromosomal SNP markers by using the R based software packages genetics and LDheatmap (Shin et al., 2006; Warnes et al., 2013; R Core Team, 2014). Genetic distances between markers in base pairs were plotted against the *r*^2^ values. The critical *r*^2^ value was set to *r*^2^ = 0.1 (Voss-Fels et al., 2015; Oyiga et al., 2017). LD decay was calculated by fitting a smooth locally weighted polynomial regression (LOESS) curve by using the software package R (R Core Team, 2014; Sannemann et al., 2015). LD decay was determined as the intersection point of the LOESS curve with the critical *r*^2^ value.

To estimate kinship matrix (K matrix) and population structure, 569 highly informative markers equally distributed on the genomes were selected (marker set described in more detail in Lehnert et al. (2017)), based on map position and polymorphism information content (PIC) value (Hildebrand et al., 1992). Rogers' distances (RD) were calculated for each pairwise genotype–genotype combination (Reif et al., 2005). For generating the K matrix, the RD matrix was converted in a standardized similarity matrix. Bayesian cluster analysis implemented in the STRUCTURE software package version 2.3.4 (Pritchard et al., 2000) and principal coordinate analysis (PCoA) using the software package DARwin 5.0 (Perrier and Jacquemoud-Collet, 2006) were conducted to determine population structure (methods are described in more detail in Lehnert et al. (2017)). Based on 15511 SNP markers and phenotypic data, GWAS was conducted for each single environment (irrigation × mycorrhizae interaction) separately and across multi environments (combined) using the software package TASSEL 4.1 (Bradbury et al., 2007). Several association models were tested to determine the most powerful model [association models are described in more detail in Lehnert et al. (2017)]. Compressed mixed linear model corrected for K matrix (CMLM+K) showed the best approximation of the expected cumulative distribution of *p*-values and the lowest mean squared difference (MSD) value (Yu et al., 2006; Stich et al., 2008). Therefore, GWAS was conducted with Tassel 4.1 by using the CMLM approach (Yu et al., 2006; Zhang et al., 2010) which also implemented the EMMA (Kang et al., 2008) and P3D (Zhang et al., 2010) algorithms to reduce computing time.

As markers in GWAS are not independent due to the assumption of LD between markers (Bush and Moore, 2012), adapted Bonferroni-Holm correction (use effective number of independent test instead of number of all tests in the denominator) was used to adjust for multiple testing (Gao et al., 2010; Johnson et al., 2010). Effective number of independent tests was calculated by performing principle component analysis for all markers of each chromosome (principal component cutoff: 0.90) by using the software package R Version 3.4.0 (R Core Team, 2014). Minimal number of principle components explaining 90% of the total variance per chromosome was summed up to estimate effective numbers of independent tests per genome (Jiang et al., 2015; Mirdita et al., 2015), resulting in a genomewide significance threshold of LOD 4.25.

Significantly associated markers were assigned to QTL regions based on the trait, the estimated LD decay [6.5 million base pairs (Mb)] and their chromosomal positions. The marker with the highest LOD value that best tags the QTL region (Maccaferri et al., 2015; Naruoka et al., 2015) was defined as peak marker (Desiderio et al., 2014; hereafter: QTL peak marker) of a chromosomal region. All significantly associated markers located within an interval of ± 6.5 Mb around the QTL peak marker were assigned to the same QTL region. The software package Circos (Krzywinski et al., 2009) was used to create the circos plots.

Identified QTL regions were compared with findings of previous studies dealing with drought stress tolerance in wheat. In the case that the previous reported QTL regions were identified by SNP markers with known flanking sequences, these sequences were remapped to the reference genome of Chinese Spring to enable the comparison between the genetic and physical map positions. If previous reported QTL regions were identified by other marker systems comparisons between studies were conducted based on chromosomes.

Genes located within a QTL region were identified based on their position on the bread wheat reference genome (IWGSC, 2018). Protein sequences of identified genes (IWGSC, 2018) were analyzed by blast analysis (NCBI blast + blastp, Camacho et al., 2009; Cock et al., 2013). Functional protein annotation was conducted by using the Blast2GO Pipeline version 2.5.0 (Conesa et al., 2005; Conesa and Götz, 2008; Götz et al., 2008; Cock et al., 2013). Gene onthology (GO) terms associated with drought, osmotic stress or interaction with symbionts (Binns et al., 2009; Li et al., 2012; Liu et al., 2015) were used to evaluate genes located within the QTL regions (Supplementary Table 2). Furthermore, QTL regions were screened for transcription factor genes (i.e., abscisic acid-responsive element binding proteins family (AREB/ABF), APETALA2/Ethylene response element binding factor family (AP2/ERF), NAC superfamily, basic leucine zipper (bZIP) family, myeloblastosis (MYB) protein family and myelocytomatosis (MYC) protein family) and genes coding for heat shock proteins (HSP) or late embryogenesis abundant (LEA) genes) which are known to be expressed in response to drought (Banerjee and Roychoudhury, 2016; reviewed in Joshi et al., 2016; reviewed in Jacob et al., 2017). Software basic default settings were used for all analyses mentioned above.

## Results

In all inoculated plants typical mycorrhizal structures were detected in roots, while non-inoculated control plants were not colonized by mycorrhizal fungi. Genotypic differences in the ability to form symbiosis were significant (*p* < 0.001) under drought stress and well watered conditions (Supplementary Table 3). Root colonization under drought stress conditions was significantly lower than root colonization under well watered conditions (Table 1). Mean root colonization of 35% and 47% was observed (Table 1) under drought stress and well watered conditions, respectively. Genotype means of root colonization ranged from 16 to 54% (drought stress) and 17 to 64% (well watered). Genotype means were normally distributed under drought stress conditions (Shapiro-Wilk, *P* = 0.91, α ≤ 0.05) and negatively skewed under well watered conditions (Shapiro-Wilk, *P* = 0.01, α ≤ 0.05; Supplementary Figures 1a,b). In general, in both irrigation regimes, a moderate range of colonization levels was observed. Under drought stress and well watered conditions, correlations between root colonization and biomass or straw yield were moderately negative, whereas no significant correlations were observed for root colonization and grain yield (Supplementary Table 4). The strongest positive correlations were observed between root colonization and number of grains per ear, both under drought stress and well watered conditions (Supplementary Table 4). Estimates of heritability for root colonization were relatively low under drought stress conditions (h^2^ = 0.30 ± 0.10) and well watered conditions (h^2^ = 0.34 ± 0.08) compared to the estimated heritability across both environments (h^2^ = 0.54 ± 0.07).

**Table 1:** Least square means (lsmean), minimum (Min), maximum (Max), and standard error (±SE) for root dry mass (RM) and root colonization by mycorrhizal fungi under drought stress and well watered conditions in the presence of mycorrhizae for 94 wheat genotypes evaluated in two years.

	**Drought stress**				**Well watered**			
	**lsmean**	**Min**	**Max**	**±SE**	**lsmean**	**Min**	**Max**	**±SE**
**Root dry mass**	2.43^a^	0.77	8.55	0.15	1.42^b^	0.24	5.81	0.10
**Root colonization**	34.73^a^	16.00	53.66	0.77	46.67^b^	16.92	63.78	0.77

*Means of the drought stress and well watered variant followed by the same letter are not significantly different at the 5% (α = 0.05) level*.

Root dry mass was assessed in all environments in 2013 and only in the mycorrhizae treated environments in 2014. Therefore, results of 2013 and 2014 were analyzed separately. Genotype means of 2013 were used to compare root dry mass under drought stress and well watered conditions in the presence and absence of mycorrhizae, whereas combined results of 2013 and 2014 were used to compare root dry mass under drought stress and well watered conditions in the presence of myccorrhizae across 2 years. In 2013, significant genotypic differences in root dry mass were detected (Supplementary Table 5). The effect of mycorrhizae, the effect of irrigation and the effect of mycorrhizae x irrigation interaction was significant (Supplementary Table 5). In general, under drought stress conditions root dry mass (2.72 g) was significantly (*p* < 0.001) increased compared to root dry mass under well watered conditions (0.77 g). Interestingly, differences between the mycorrhizal (2.18 g) and non-mycorrhizal treatment (3.25 g) were significant (*p* < 0.001) under drought stress condition, however, root dry mass was not affected by mycorrhizae under well watered conditions (myco +: 0.77 g, myco -: 0.77 g). Combined phenotypic data of 2013 and 2014 confirm the results of 2013. Root dry mass increased significantly (*p* < 0.001) under drought stress conditions compared to root dry mass under well watered conditions in the presence of mycorrhizae (Table 1). The correlation between root dry mass and root colonization under drought stress conditions and well watered conditions was negative (phenotypic correlation coefficient, Pearson; r_p_ = −0.22 and r_p_ = −0.38, *p* = 0.05) pointing to a reduced colonization of genotypes with extensive root systems. Estimated h^2^ was moderate under drought stress and well watered conditions (Table 2).

**Table 2:** Estimated heritabilities (h^2^) of aboveground biomass (BM), number of ears per plant (EN), number of grains per ear (GN), grain yield (GY), straw yield (SY), thousand grain weight (TGW), root dry mass (RM), and root colonization by mycorrhizal fungi under drought stress and well watered conditions in the presence (myco +) and absence (myco −) of mycorrhizae and across all environments (combined) for 94 wheat genotypes evaluated in 2 years.

		**Drought stress**		**Well watered**		**Combined**
		**myco +**	**myco −**	**myco +**	**myco −**	
	**Aboveground biomass**	0.73 ± 0.06	0.68 ± 0.07	0.66 ± 0.07	0.77 ± 0.05	0.92 ± 0.02
	**Number of ears per plant**	0.47 ± 0.11	0.67 ± 0.07	0.38 ± 0.13	0.58 ± 0.09	0.86 ± 0.03
	**Number of grains per ear**	0.78 ± 0.05	0.82 ± 0.04	0.81 ± 0.04	0.82 ± 0.04	0.86 ± 0.03
**h**^2^ ± **SE**	**Grain yield**	0.63 ± 0.06	0.70 ± 0.05	0.55 ± 0.07	0.47 ± 0.09	0.77 ± 0.05
	**Straw yield**	0.95 ± 0.01	0.94 ± 0.01	0.90 ± 0.02	0.93 ± 0.01	0.97 ± 0.01
	**Thousand grain weight**	0.85 ± 0.03	0.77 ± 0.05	0.75 ± 0.05	0.87 ± 0.03	0.93 ± 0.01
	**Root dry mass**	0.69 ± 0.06	0.69 ± 0.06^a^	0.54 ± 0.10	0.70 ± 0.23^a^	0.76 ± 0.05
	**Root colonization**	0.30 ± 0.10		0.34 ± 0.08		0.54 ± 0.07

a *Repeatibility was calculated instead of h^2^*.

**Figure 1:**
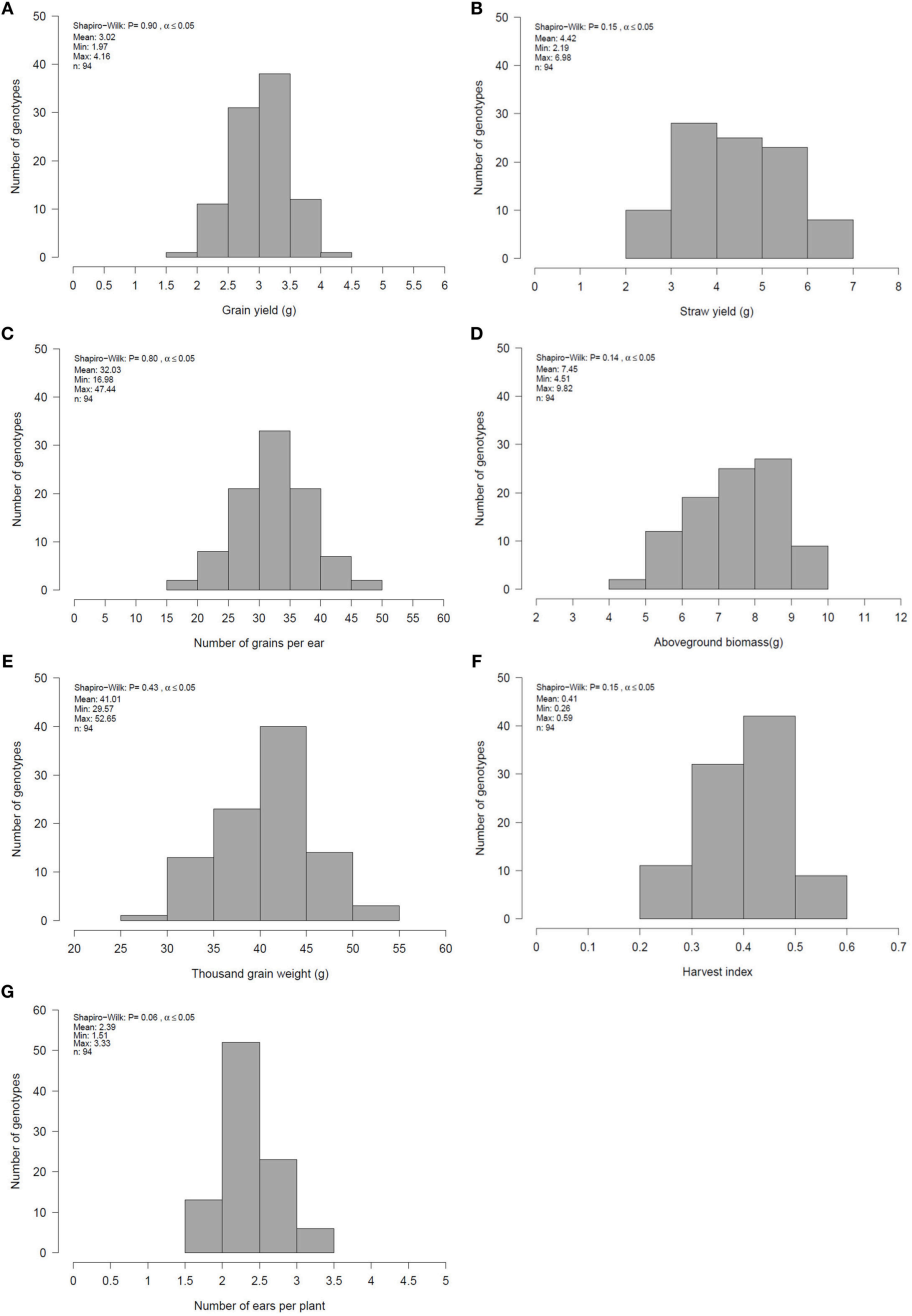
Mean, minimum (Min), maximum (Max) and distribution of genotype means for **(A)** grain yield (GY), **(B)** straw yield (SY), **(C)** number of grain per ear (GN), **(D)** aboveground biomass (BM), **(E)** thousand grain weight (TGW), **(F)** harvest index (HI) and **(G)** number of ears per plant (EN) for 94 genotypes across all environments evaluated in 2 years.

Single and combined analysis of variance revealed a significant effect of the genotype on all yield and yield related traits (Supplementary Tables 3,5). The effect of irrigation and the interaction effect of mycorrhizae x irrigation was also significant for all traits (*p* < 0.05; Supplementary Table 5). However, the interaction effect of genotype x environment (genotype x mycorrhizae × irrigation) was not significant for all traits (Supplementary Table 5). Genotype means across all environments (combined) were normally distributed for all traits (Figures 1A–G), pointing to a large genotypic variation. For all traits, estimated h^2^ across all environments was high (h^2^ > 0.70) and ranged between 0.77 (grain yield) and 0.97 (straw yield; Table 2). Traits were weakly to highly correlated, whereby the highest positive or negative correlations were r_p_ = −0.54 (GN vs. EN) or r_p_ = 0.93 (SY vs. BM), respectively (Table 3). Estimates of r_p_ between grain yield and yield components were weak to moderate and positive. Grain yield was most affected by number of grains per ear (Table 3). Under drought stress and well watered conditions, estimates of h^2^ ranged between 0.47 (EN) and 0.95 (SY) or 0.38 (EN) and 0.93 (SY), respectively. In general, estimates of h^2^ were higher under drought stress conditions compared to well watered conditions for BM, EN, SY, TGW, and RM in the presence of mycorrhizae and for EN, GN, and SY in the absence of mycorrhizae (Table 2). These estimates provide a first approximation of h^2^, but further multiannual experiments are necessary to validate these findings. Correlations between environments were moderate to strong and positive for all traits (Supplementary Table 4).

**Table 3:** Phenotypic correlation coefficient, (phenotypic correlation coefficient, Pearson) between traits across environments.

**Variable**	**Aboveground biomass**	**Ears per plant**	**Grains per ear**	**Grain yield**	**Straw yield**	**Thousand grain weight**
**Aboveground biomass**	–	*n*.*s*.	*n*.*s*.	^*^	^*^	^*^
**Number of ears per plant**	0.17	–	^*^	*n*.*s*.	n.s.	^*^
**Number of grains per ear**	−0.07	−0.54	–	^*^	^*^	^*^
**Grain yield**	0.32	0.13	0.46	–	n.s.	^*^
**Straw yield**	0.93	0.13	−0.24	−0.04	–	n.s.
**Thousand grain weight**	0.23	−0.28	−0.26	0.24	0.16	–

* *Significant (α = 0.05); ns, not significant*.

**Figure 2:**
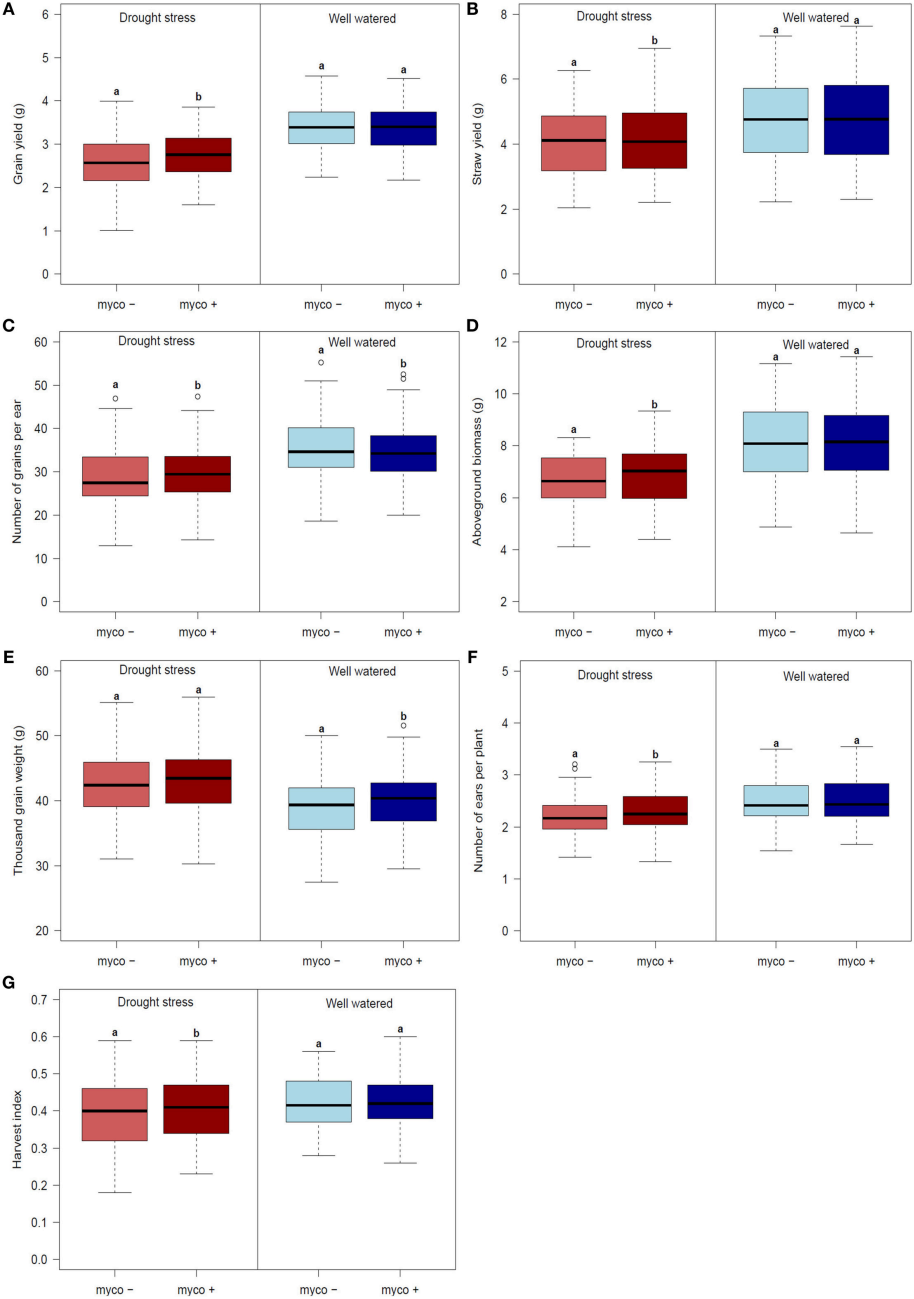
Boxplots of genotype means for **(A)** grain yield (GY), **(B)** straw yield (SY), **(C)** number of grain per ear (GN), **(D)** aboveground biomass (BM), **(E)** thousand grain weight (TGW), **(F)** harvest index (HI) and **(G)** number of ears per plant (EN) for 94 genotypes under drought stress (left) and well watered (right) conditions in the presence (myco +) and absence (myco −) of mycorrhizae evaluated in 2 years. Means of the mycorrhizal and non-mycorrhizal treatment followed by the same letter under drought stress or well watered conditions, respectively, are not significantly different at the 5% (α = 0.05) level.

Differences in the performance between drought stress and well watered conditions were significant for all traits (Table 4). Drought stress caused a reduction of GY, GN, BM, SY, and EN (listed in decreasing order) in the presence and absence of mycorrhizae, while TGW increased (summarized in Figures 2A–G, Table 4). Reductions due to drought ranged from 9% (EN) to 19% (GY) in the presence of mycorrhizae and 12% (EN) to 24% (GY) in the absence of mycorrhizae. Under drought stress conditions, reduction in GY, SY, GN, EN, and BM was significantly lower in mycorrhizae treated plants compared to non-treated plants (Figure 2). Mycorrhizae treatment increased GY, EN, GN and TGW by 7, 5, 3, and 1%, respectively under drought stress conditions compared to the non-mycorrhizae treatment. No significant differences in TGW were detected between the mycorrhizae and non-mycorrhizae treatment under drought stress conditions (Figure 2, Table 4). Furthermore, SY, GY, EN, and BM were not significantly influenced by mycorrhizae under well watered conditions, whereas GN and TGW were significantly negatively or positively, respectively, influenced by mycorrhizae (Figure 2).

**Table 4:** Least square means (lsmean), minimum (Min), maximum (Max) and standard error (±SE) for aboveground biomass (BM), number of ears per plant (EN), number of grains per ear (GN), grain yield (GY), straw yield (SY), thousand grain weight (TGW), and harvest index (HI) under drought stress and well watered conditions in the presence (myco +) and absence (myco −) of mycorrhizae for 94 wheat genotypes evaluated in two years.

		**Drought stress**				**Well watered**			
		**lsmean**	**Min**	**Max**	**±SE**	**lsmean**	**Min**	**Max**	**±SE**
**myco** +	**Aboveground biomass**	6.89^a^	4.41	9.34	0.11	8.01^b^	4.65	11.42	0.15
	**Number of ears per plant**	2.31^a^	1.33	3.25	0.04	2.53^b^	1.67	3.54	0.04
	**Number of grains per ear**	29.42^a^	14.32	47.43	0.69	34.77^b^	19.96	52.45	0.67
	**Grain yield**	2.75^a^	1.60	3.86	0.05	3.38^b^	2.17	4.51	0.05
	**Straw yield**	4.14^a^	2.20	6.95	0.11	4.69^b^	2.29	7.64	0.13
	**Thousand grain weight**	42.70^a^	30.30	55.97	0.57	39.91^b^	29.48	51.60	0.49
	**Harvest index**	0.40^a^	0.23	0.59	0.01	0.42^b^	0.26	0.60	0.01
**myco** −	**Aboveground biomass**	6.61^a^	4.12	8.31	0.11	8.12^b^	4.87	11.1521	0.16
	**Number of ears per plant**	2.21^a^	1.42	3.21	0.04	2.50^b^	1.54	3.50	0.04
	**Number of grains per ear**	28.53^a^	12.94	46.91	0.71	35.66^b^	18.61	55.21	0.71
	**Grain yield**	2.56^a^	1.01	4.00	0.06	3.38^b^	2.24	4.58	0.05
	**Straw yield**	4.04^a^	2.04	6.27	0.11	4.71^b^	2.23	7.33	0.14
	**Thousand grain weight**	42.45^a^	31.03	55.16	0.52	38.79^b^	27.44	50.07	0.51
	**Harvest index**	0.39^a^	0.18	0.59	0.01	0.42^b^	0.28	0.56	0.01

*Means of the drought stress and well watered variant followed by the same letter are not significantly different at the 5% level (α = 0.05), respectively*.

Two drought stress susceptibility indices (IT, STI) were calculated to evaluate drought stress tolerance of each genotype in the presence and absence of mycorrhizae, respectively. For IT and STI of yield, genotype means showed a continuous distribution in the presence and absence of mycorrhizae, indicating that genotypic variation in drought stress tolerance is present (data not shown). As already indicated, drought stress tolerance (IT and STI) was significantly higher in the presence of mycorrhizae as in the absence of mycorrhizae for SY, GY, GN, and BM, pointing to a significantly increased drought stress tolerance in the presence of mycorrhizae (Supplementary Table 6).

**Figure 3:**
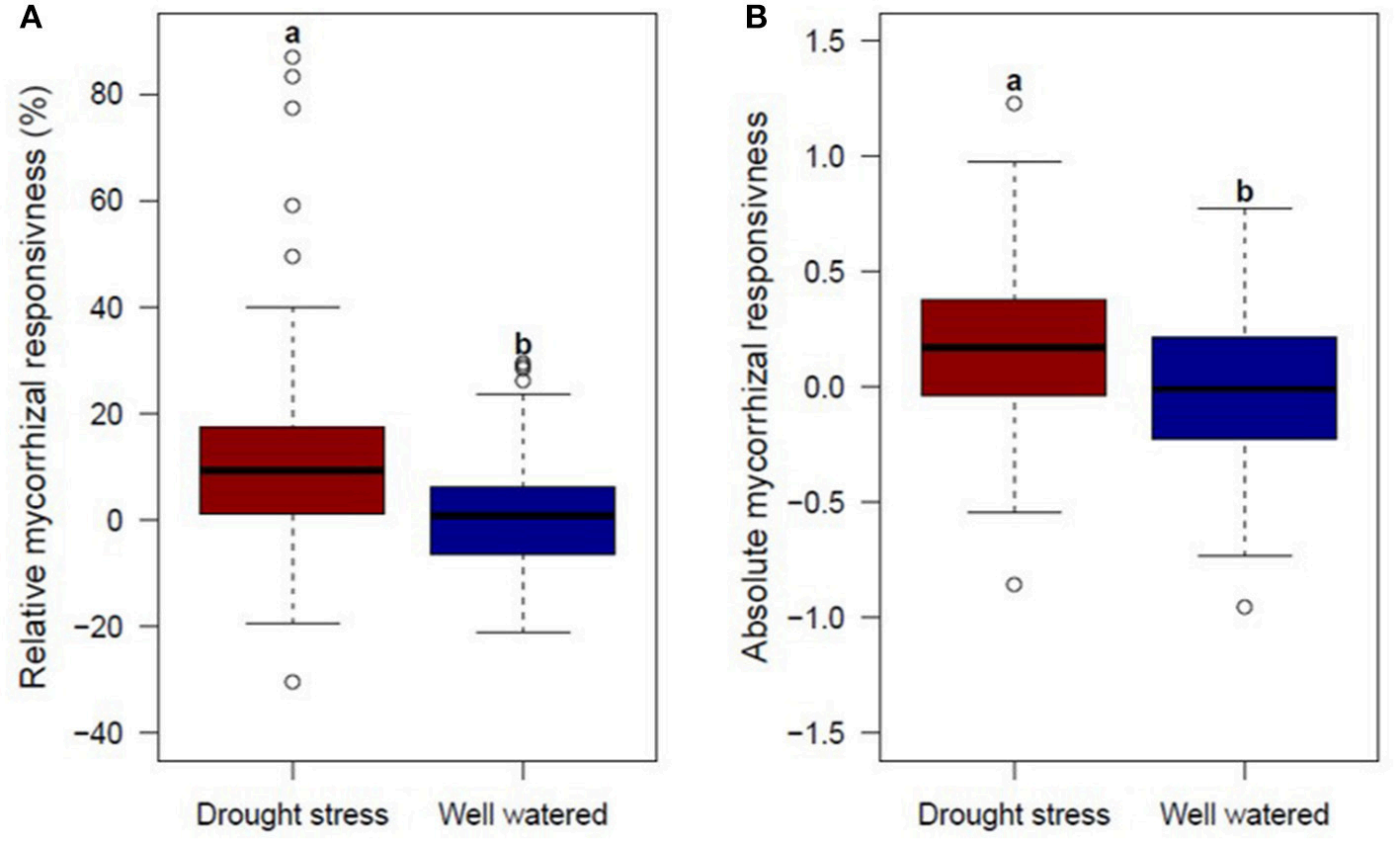
Boxplots of genotype means for **(A)** relative mycorrhizal responsivness (MR) and **(B)** absolute mycorrhizal responsivness (R), for 94 genotypes under drought stress (left) and well watered (right) conditions evaluated in 2 years. Means followed by the same letter are not significantly different at the 5% (α = 0.05) level.

To identify genotypes, which use the mycorrhizae symbiosis effectively, relative mycorrhizal responsiveness and absolute mycorrhizal responsiveness were calculated under drought stress and well watered conditions. Both mean relative and absolute mycorrhizal responsiveness for GY were significantly higher under drought stress conditions compared to responsiveness under well watered conditions (Figure 3, Supplementary Table 7). However, not all genotypes responded positively to mycorrhizae. Under drought stress and well watered conditions, two thirds and half of the genotypes analyzed were positively influenced by mycorrhizae (Figure 3). Results give hint to an increased impact of mycorrhizae under drought stress conditions due to limited water availability. Correlations between relative or absolute mycorrhizal responsiveness for GY and root colonization by mycorrhizal fungi were weakly positive but not significant under drought stress (r_p_ = 0.20 and r_p_ = 0.08, respectively) and well watered conditions (r_p_ = 0.17 and r_p_ = 0.04, respectively).

Relative and absolute responsiveness (for GY) were significantly negatively correlated with GY under drought stress conditions in the absence of mycorrhizae (r_p_ = −0.62 and r_p_ = −0.50, respectively; *p* ≤ 0.001), pointing out that genetic variation in response to mycorrhizae is linked to differences in the performance in the absence of mycorrhizae. In order to determine specific genetic variation regarding the response to mycorrhizae, which can be effectively used in plant breeding and which is not due to genotypic differences in the performance in the absence of mycorrhizae, linear regression analyses were conducted.

Linear regression analysis of GY under drought stress conditions in the presence of mycorrhizae against GY under drought stress conditions in the absence of mycorrhizae was performed. GY under drought stress conditions in the presence of mycorrhizae was significantly positively correlated (r_p_ = 0.81, *p* ≤ 0.001) with GY under drought stress conditions in the absence of mycorrhizae, pointing to a weak specific variation (1-r_p_ = 0.19) in response to mycorrhizae in the investigated genotype set (Supplementary Figure 2). Linear regression was estimated to be y = 0.71x + 0.94 with R^2^ = 0.66 (Supplementary Figure 2). Genotypes were assigned to groups according to the year of release and grouping by the software STRUCTURE. In both grouping scenarios, different genotype groups fell along the same regression line, pointing out that a positive response to mycorrhizae is not associated with genotypes of one group and that plant breeding did not result in reduced response to mycorrhizae in modern wheat cultivars (Supplementary Figure 2). Deviation (δ) from the regression line of GY under drought stress conditions in the presence of mycorrhizae against GY under drought stress conditions in the absence of mycorrhizae was calculated for each genotype to identify genotypes with highest positive specific variation. In total, 49 % of genotypes showed positive deviations from the regression line and are therefore revealing a beneficial specific variation in response to mycorrhizae (Supplementary Figure 3).

Genotyping of the 94 wheat genotypes resulted in a raw data set of 81587 SNP markers. Quality filtering of the raw marker data set resulted in a filtered marker set of 15511 informative, polymorphic and mapped markers. In total, 66076 markers were excluded, due to minor allele frequency <5% [28277; including monomorphic markers (23536)], missing values >10% (9911), very poor quality (130; inconsistent data in replicated controls) or no unique map position on the bread wheat reference genome (27277, IWGSC, 2018). Based on 569 highly informative markers, distributed equally on the wheat chromosomes, the K matrix and the population structure matrix were calculated. Results of Bayesian clustering analysis (optimal number of k = 2, membership coefficient ≥0.7) and PCoA (PCo1 = 8.19%, PCo2 = 5.61%) indicate a weak population structure [results are described in more detail in Lehnert et al. (2017)]. Genotypic and phenotypic data were used to conduct GWAS. The K model including only the K matrix as correction for relatedness turned out to be the most appropriate model for control false positives (data not shown). Therefore, a compressed mixed linear model with the K matrix as correction for relatedness was fitted in Tassel 4.1 to conduct GWAS. The LD decay was calculated for each chromosome separately and across all 21 chromosomes. The LD across all chromosomes decayed at 6,447,100 bp, whereas LD decay calculated for each chromosome separately ranged between 1,192,633 bp (chromosome 6D) and 15,810,649 bp (chromosome 2D) (Supplementary Figure 4).

In total, 187 marker trait associations (MTAs) significantly associated (LOD ≥ 4.25) with traits under investigation were identified (Supplementary Table 8). Significantly, associated MTAs were assigned to 84 QTL regions on 16 chromosomes, whereby the number of significantly associated markers per QTL region ranged between 1 and 13 (Table 5, Supplementary Table 8). Most QTL regions were detected on chromosome 6A (17) and 3D (9), whereas no QTL was identified on chromosome 4B, 4D, 5A, 5D, and 7A (Table 5). Most QTL are associated with SY (23). In total, 57, 13, and 14 QTL regions were identified by single environment GWAS, multi environment (combined) GWAS and GWAS for drought stress or mycorrhizae responsiveness associated traits, respectively. Out of the QTL regions identified by single environment GWAS, 30 QTL region were identified under drought stress conditions (myco +: 16; myco -: 14), whereas 27 QTL regions were detected under well watered conditions (myco +: 21; myco -: 6; Table 5). Furthermore, multi trait QTL regions—associated with more than on trait—were identified on chromosome 1B, 1D, 3D, and 6A.

**Table 5:** Quantitative trait loci (QTL) regions and QTL peak marker (LOD ≥ 4.25) associated with traits under consideration.

**Environment**	**Trait**	**Mycorrhization**	**Irrigation**	**QTL**	**Number of markers**	**Peakmarker**	**Chromosome**	**Position (bp)**	**R^2^ (%)**	**PIC**	**LOD**	**SNP**	**MAF**	**Effect**
**single**	**Aboveground biomass**	+	**DS**	*QTL_BMmyc25_1D*	*1*	BS00022178_51	**1D**	**16612833**	6.91	0.36	4.57	T/C	0.36	0.63
**single**	**Aboveground biomass**	+	**DS**	*QTL_BMmyc25_2A*	*2*	BS00086329_51	**2A**	**711077642**	6.47	0.37	4.33	G/T	0.43	0.53
**single**	**Aboveground biomass**	+	**DS**	*QTL_BMmyc25_4A*	*3*	Ra_c45147_1600	**4A**	**492891477**	8.00	0.32	4.41	A/G	0.28	−0.93
**single**	**Aboveground biomass**	+	**WW**	*QTL_BMmyc75_4A*	*2*	BS00065509_51	**4A**	**590123186**	3.94	0.21	4.88	A/C	0.14	2.02
**single**	**Aboveground biomass**	−	**DS**	*QTL_BM25_4A*	*2*	BS00064810_51	**4A**	**595376414**	7.02	0.20	4.59	G/A	0.13	1.47
**single**	**Number of grains per ear**	+	**DS**	*QTL_GNmyc25_2B*	*3*	wsnp_Ex_c5123_9089025	**2B**	**696678792**	8.96	0.21	4.26	A/G	0.14	−8.41
**single**	**Number of grains per ear**	+	**DS**	*QTL_GNmyc25_7D*	*1*	BobWhite_c11327_185	**7D**	**19405530**	9.35	0.19	4.41	T/C	0.12	−9.39
**single**	**Number of grains per ear**	+	**WW**	*QTL_GNmyc75_2B*	*1*	Kukri_c55996_63	**2B**	**765069399**	13.87	0.26	6.01	C/T	0.19	7.23
**single**	**Number of grains per ear**	+	**WW**	*QTL_GNmyc75_2D*	*1*	RAC875_c5673_1209	**2D**	**622382798**	11.81	0.28	5.25	G/T	0.21	6.54
**single**	**Number of grains per ear**	−	**DS**	*QTL_GN25_1D*	*1*	BS00096470_51	**1D**	**5357586**	8.58	0.29	4.30	T/C	0.23	7.21
**single**	**Number of grains per ear**	−	**DS**	*QTL_GN25_2B*	*3*	wsnp_Ex_c5123_9089025	**2B**	**696678792**	8.64	0.21	4.33	A/G	0.14	−8.63
**single**	**Grain yield**	+	**WW**	*QTL_GYmyc75_6A*	*2*	wsnp_Ex_c20457_29526403	**6A**	**596422223**	3.51	0.19	4.57	C/T	0.12	−0.72
**single**	**Grain yield**	−	**DS**	*QTL_GY25_1D*	*1*	BS00096470_51	**1D**	**5357586**	6.29	0.29	4.67	T/C	0.23	0.62
**single**	**Grain yield**	−	**DS**	*QTL_GY25_3D*	*1*	BS00065107_51	**3D**	**499582732**	7.66	0.22	4.73	A/C	0.14	1.65
**single**	**Straw yield**	+	**DS**	*QTL_SYmyc25_5B*	*2*	RFL_Contig5739_641	**5B**	**531538658**	9.16	0.28	4.56	A/G	0.21	−0.76
**single**	**Straw yield**	+	**DS**	*QTL_SYmyc25_6A-1*	*1*	BS00027313_51	**6A**	**151492080**	8.85	0.33	4.43	C/T	0.29	0.89
**single**	**Straw yield**	+	**DS**	*QTL_SYmyc25_6A-2*	*1*	BS00022120_51	**6A**	**396301420**	9.62	0.37	4.75	T/C	0.40	0.84
**single**	**Straw yield**	+	**WW**	*QTL_SYmyc75_6A-1*	*3*	IAAV6992	**6A**	**401890392**	5.62	0.35	4.38	T/C	0.34	1.16
**single**	**Straw yield**	+	**WW**	*QTL_SYmyc75_6A-2*	*4*	wsnp_Ex_c590_1176006	**6A**	**410580347**	5.62	0.35	4.38	T/C	0.34	1.16
**single**	**Straw yield**	+	**WW**	*QTL_SYmyc75_6A-3*	*13*	BS00086046_51	**6A**	**425277756**	5.62	0.35	4.38	C/T	0.34	1.16
**single**	**Straw yield**	+	**WW**	*QTL_SYmyc75_6A-4*	*12*	BS00099290_51	**6A**	**438579488**	5.62	0.35	4.38	C/T	0.34	1.16
**single**	**Straw yield**	+	**WW**	*QTL_SYmyc75_6B*	*1*	wsnp_Ku_c3354_6228863	**6B**	**454509173**	5.62	0.35	4.38	A/G	0.34	1.16
**single**	**Straw yield**	+	**WW**	*QTL_SYmyc75_6D*	*1*	BS00064203_51	**6D**	**288554045**	5.62	0.35	4.38	A/G	0.34	1.16
**single**	**Straw yield**	−	**DS**	*QTL_SY25_6A*	*1*	BS00022120_51	**6A**	**396301420**	8.66	0.37	4.28	T/C	0.40	0.88
**single**	**Straw yield**	−	**WW**	*QTL_SY75_1A*	*1*	wsnp_Ex_c9722_16062937	**1A**	**519469969**	8.87	0.31	4.53	C/T	0.26	0.68
**single**	**Straw yield**	−	**WW**	*QTL_SY75_1B*	*1*	Kukri_c18230_1633	**1B**	**664787622**	8.90	0.35	4.54	T/G	0.35	0.63
**single**	**Straw yield**	−	**WW**	*QTL_SY75_7B*	*1*	Kukri_c51296_438	**7B**	**565911676**	9.12	0.30	4.64	T/C	0.24	0.77
**single**	**Thousand grain weight**	+	**WW**	*QTL_TGWmyc75_3B*	*1*	Tdurum_contig21756_137	**3B**	**721071723**	5.40	0.37	4.29	A/G	0.46	−4.18
**single**	**Thousand grain weight**	−	**DS**	*QTL_TGW25_6D*	*1*	D_contig10996_530	**6D**	**463743339**	8.57	0.35	4.47	A/G	0.35	−2.22
**single**	**Harvest index**	+	**DS**	*QTL_HImyc25_1B-1*	1	Tdurum_contig58525_304	**1B**	**14152715**	7.81	0.198	4.65	C/A	0.128	0.1653
**single**	**Harvest index**	+	**DS**	*QTL_HImyc25_1B-2*	1	Kukri_c18230_1633	**1B**	**664787622**	8.00	0.352	4.74	T/G	0.351	−0.122
**single**	**Harvest index**	+	**DS**	*QTL_HImyc25_3B*	1	RFL_Contig5246_1091	**3B**	**447287184**	12.34	0.12	6.01	A/G	0.069	0.6107
**single**	**Harvest index**	+	**DS**	*QTL_HImyc25_4A-1*	1	BobWhite_c1907_124	**4A**	**216145861**	8.33	0.279	4.91	C/A	0.213	0.1475
**single**	**Harvest index**	+	**DS**	*QTL_HImyc25_4A-2*	1	wsnp_Ex_c12812_20324273	**4A**	**605729749**	7.56	0.331	4.52	A/G	0.298	0.1164
**single**	**Harvest index**	+	**DS**	*QTL_HImyc25_5B*	1	wsnp_JD_rep_c63083_40243538	**5B**	**541612773**	8.55	0.362	4.26	C/T	0.388	0.1319
**single**	**Harvest inde**x	+	**DS**	*QTL_HImyc25_6B*	2	Tdurum_contig50121_249	**6B**	**28153575**	8.33	0.262	4.91	G/A	0.191	−0.169
**single**	**Harvest index**	+	**DS**	*QTL_HImyc25_6D*	1	Kukri_c10377_112	**6D**	**16295376**	7.34	0.314	4.41	G/A	0.266	−0.143
**single**	**Harvest index**	+	**WW**	*QTL_HImyc75_2A*	1	wsnp_RFL_Contig2744_2471775	**2A**	**69352365**	5.63	0.301	4.35	C/T	0.245	−0.145
**single**	**Harvest index**	+	**WW**	*QTL_HImyc75_2B-1*	11	BS00021675_51	**2B**	**104792516**	5.63	0.301	4.35	C/T	0.245	−0.145
**single**	**Harvest index**	+	**WW**	*QTL_HImyc75_2B-2*	11	BS00022486_51	**2B**	**121371060**	5.63	0.301	4.35	A/C	0.245	−0.145
**single**	**Harvest index**	+	**WW**	*QTL_HImyc75_2B-3*	1	BS00083078_51	**2B**	**131130470**	5.63	0.301	4.35	G/A	0.245	−0.145
**single**	**Harvest index**	+	**WW**	*QTL_HImyc75_2D-1*	1	IAAV666	**2D**	**62958775**	5.54	0.252	4.29	G/T	0.181	−0.154
**single**	**Harvest index**	+	**WW**	*QTL_HImyc75_2D-2*	1	IAAV2480	**2D**	**77131602**	5.63	0.301	4.35	T/C	0.245	−0.145
**single**	**Harvest index**	+	**WW**	*QTL_HImyc75_3A*	1	Excalibur_c29769_81	**3A**	**662597671**	5.51	0.361	4.27	C/T	0.383	0.1122
**single**	**Harvest index**	+	**WW**	*QTL_HImyc75_6B*	1	Kukri_c25377_240	**6B**	**174568619**	6.57	0.34	4.95	C/T	0.319	0.1399
**single**	**Harvest index**	−	**DS**	*QTL_HI25_3B*	1	BS00085434_51	**3B**	**750357915**	7.78	0.204	4.41	C/T	0.133	0.857
**single**	**Harvest index**	−	**DS**	*QTL_HI25_3D*	1	BS00065107_51	**3D**	**499582732**	8.40	0.216	4.75	A/C	0.144	0.5919
**single**	**Harvest index**	−	**WW**	*QTL_HI75_3B*	1	BS00085434_51	**3B**	**750357915**	5.83	0.204	4.32	C/T	0.133	0.4038
**single**	**Harvest index**	−	**WW**	*QTL_HI75_3D*	1	BS00065107_51	**3D**	**499582732**	5.91	0.216	4.37	A/C	0.144	0.2272
**single**	**Harvest index**	−	**WW**	*QTL_HI75_5B*	1	Kukri_c13048_341	**5B**	**606026241**	5.03	0.368	4.49	C/T	0.415	0.1467
**single**	**Root dry mass**	+	**WW**	*QTL_RMmyc75_3B*	*1*	Excalibur_c21751_421	**3B**	**655310004**	5.92	0.14	4.43	A/G	0.09	−1.38
**single**	**Root dry mass**	+	**WW**	*QTL_RMmyc75_7B*	*1*	Excalibur_c41549_276	**7B**	**243566481**	5.74	0.17	4.32	C/T	0.11	1.16
**single**	**Root dry mass**	−	**DS**	*QTL_RM25_3A*	*2*	tplb0053a24_1247	**3A**	**8590685**	29.60	0.27	5.17	C/T	0.20	−1.92
**single**	**Root dry mass**	−	**DS**	*QTL_RM25_3B*	*2*	GENE-1785_118	**3B**	**760136494**	24.77	0.34	4.41	C/T	0.32	−1.78
**single**	**Root dry mass**	−	**DS**	*QTL_RM25_3D-1*	*2*	Ra_c10284_405	**3D**	**209687062**	24.45	0.34	4.36	C/T	0.31	−1.72
**single**	**Root dry mass**	−	**DS**	*QTL_RM25_3D-2*	*3*	wsnp_Ku_c25527_35493338	**3D**	**610348624**	24.43	0.26	4.36	T/C	0.19	−2.34
**single**	**Root dry mass**	−	**DS**	*QTL_RM25_3D-3*	*5*	wsnp_Ex_c12963_20529964	**3D**	**612902535**	24.53	0.34	4.37	G/T	0.32	−1.72
**combined**	**Aboveground biomass**			*QTL_BMc_4A*	*2*	BS00064810_51	**4A**	**595376414**	3.78	0.20	4.62	G/A	0.13	1.65
**combined**	**Aboveground biomass**			*QTL_BMc_6A*	*1*	BS00027313_51	**6A**	**151492080**	3.54	0.33	4.38	C/T	0.29	1.26
**combined**	**Aboveground biomass**			*QTL_BMc_6A-2*	*1*	BS00022120_51	**6A**	**396301420**	3.51	0.37	4.34	T/C	0.40	1.08
**combined**	**Number of grains per ear**			*QTL_GNc_2B*	*3*	wsnp_Ex_c5123_9089025	**2B**	**696678792**	8.84	0.21	4.26	A/G	0.14	−7.41
**combined**	**Straw yield**			*QTL_SYc_6A-1*	*1*	BS00027313_51	**6A**	**151492080**	6.74	0.33	4.34	C/T	0.29	1.10
**combined**	**Straw yield**			*QTL_SYc_6A-2*	*2*	BS00022120_51	**6A**	**396301420**	7.11	0.37	4.54	T/C	0.40	0.97
**combined**	**Straw yield**			*QTL_SYc_6A-3*	*1*	wsnp_Ku_c8394_14267750	**6A**	**402952170**	6.88	0.35	4.42	G/A	0.34	1.05
**combined**	**Straw yield**			*QTL_SYc_6A-4*	*4*	wsnp_Ex_c590_1176006	**6A**	**410580347**	6.88	0.35	4.42	T/C	0.34	1.05
**combined**	**Straw yield**			*QTL_SYc_6A-5*	*13*	BS00086046_51	**6A**	**425277756**	6.88	0.35	4.42	C/T	0.34	1.05
**combined**	**Straw yield**			*QTL_SYc_6A-6*	*12*	BS00099290_51	**6A**	**438579488**	6.88	0.35	4.42	C/T	0.34	1.05
**combined**	**Straw yield**			*QTL_SYc_6B*	*1*	wsnp_Ku_c3354_6228863	**6B**	**454509173**	6.88	0.35	4.42	A/G	0.34	1.05
**combined**	**Straw yield**			*QTL_SYc_6D*	*1*	BS00064203_51	**6D**	**288554045**	6.88	0.35	4.42	A/G	0.34	1.05
**combined**	**Root dry mass**			*QTL_RMc_3D*	*1*	Kukri_c50527_241	**3D**	**3299007**	25.17	0.37	4.48	C/T	0.43	−1.85
**STI**	**Aboveground biomass**	+		*QTL_STI_BMmyc_4A*	*1*	BS00065509_51	**4A**	**590123186**	8.14	0.21	4.33	A/C	0.14	0.34
**STI**	**Aboveground biomass**	−		*QTL_STI_BM_1D*	*1*	BS00022178_51	**1D**	**16612833**	9.18	0.36	4.29	T/C	0.36	0.24
**STI**	**Number of ears per plant**	+		*QTL_STI_ENmyc_7D*	*1*	Tdurum_contig59690_2020	**7D**	**2327197**	9.36	0.10	4.46	G/A	0.05	−0.62
**STI**	**Number of ears per plant**	−		*QTL_STI_EN_7D*	*1*	Tdurum_contig59690_2020	**7D**	**2327197**	9.70	0.10	4.50	G/A	0.05	−0.60
**STI**	**Number of grains per ear**	−		*QTL_STI_GN_2B*	*3*	wsnp_Ex_c5123_9089025	**2B**	**696678792**	9.74	0.21	4.47	A/G	0.14	−0.42
**IT**	**Grain yield**	−		*QTL_IT_GY_3D*	*1*	BS00065107_51	**3D**	**499582732**	12.42	0.22	4.74	A/C	0.14	38.84
**STI**	**Grain yield**	+		*QTL_STI_GYmyc_6A*	*2*	wsnp_Ex_c20457_29526403	**6A**	**596422223**	11.03	0.19	5.26	C/T	0.12	−0.37
**IT**	**Straw yield**	+		*QTL_IT_SYmyc_1D*	*1*	D_contig13475_402	**1D**	**20045166**	7.53	0.20	4.25	G/A	0.13	−6.59
**IT**	**Straw yield**	−		*QTL_IT_SY_2A*	*1*	BS00021706_51	**2A**	**21261871**	12.05	0.35	4.39	T/C	0.34	11.09
**IT**	**Thousand grain weight**	+		*QTL_IT_TGWmyc_7B*	*2*	wsnp_Ex_c9909_16316813	**7B**	**36489676**	9.30	0.26	4.54	C/T	0.19	7.88
**MR**	**Number of grains per ear**		**DS**	*QTL_MR_GN25_7D*	*1*	RAC875_c19631_269	**7D**	**6694492**	8.49	0.16	4.41	T/C	0.10	−24.22
**R**	**Number of grains per ear**		**DS**	*QTL_R_GN25_7D*	*1*	RAC875_c19631_269	**7D**	**6694492**	19.98	0.16	4.39	T/C	0.10	−6.39
**δ**	**Number of grains per ear**		**DS**	*QTL_δ_GN25_7D*	*1*	RAC875_c19631_269	**7D**	**6694492**	20.39	0.16	4.47	T/C	0.10	−5.92
**MR**	**Grain yield**		**DS**	*QTL_MR_GY25_3D*	*1*	BS00065107_51	**3D**	**499582732**	10.96	0.22	4.29	A/C	0.14	−72.67

*DS, drought stress conditions; WW, well watered conditions; STI and IT, drought stress susceptibility indices; MR, relative mycorrhizal responsivness; R, abosolute mycorrhizal responsivness; δ, deviation from the regression line; R^2^, variance explained by marker in %; PIC, polymorphism information content of each marker; LOD, -log10 (p-value); MAF, minor allele frequency of each marker; Effect, allelic effect*.

Five QTL regions on chromosomes 3A, 3B, and 3D were found to be associated with RM under drought stress conditions in the absence of mycorrhizae, whereas another region on chromosome 3D showed significant association with RM across all mycorrhizae treated environments (Table 5, Supplementary Table 8). Furthermore, two chromosomal regions on chromosomes 3B and 7B were identified associated with RM under well watered conditions in the presence of mycorrhizae (Table 5, Supplementary Tables 5, 8).

**Figure 4:**
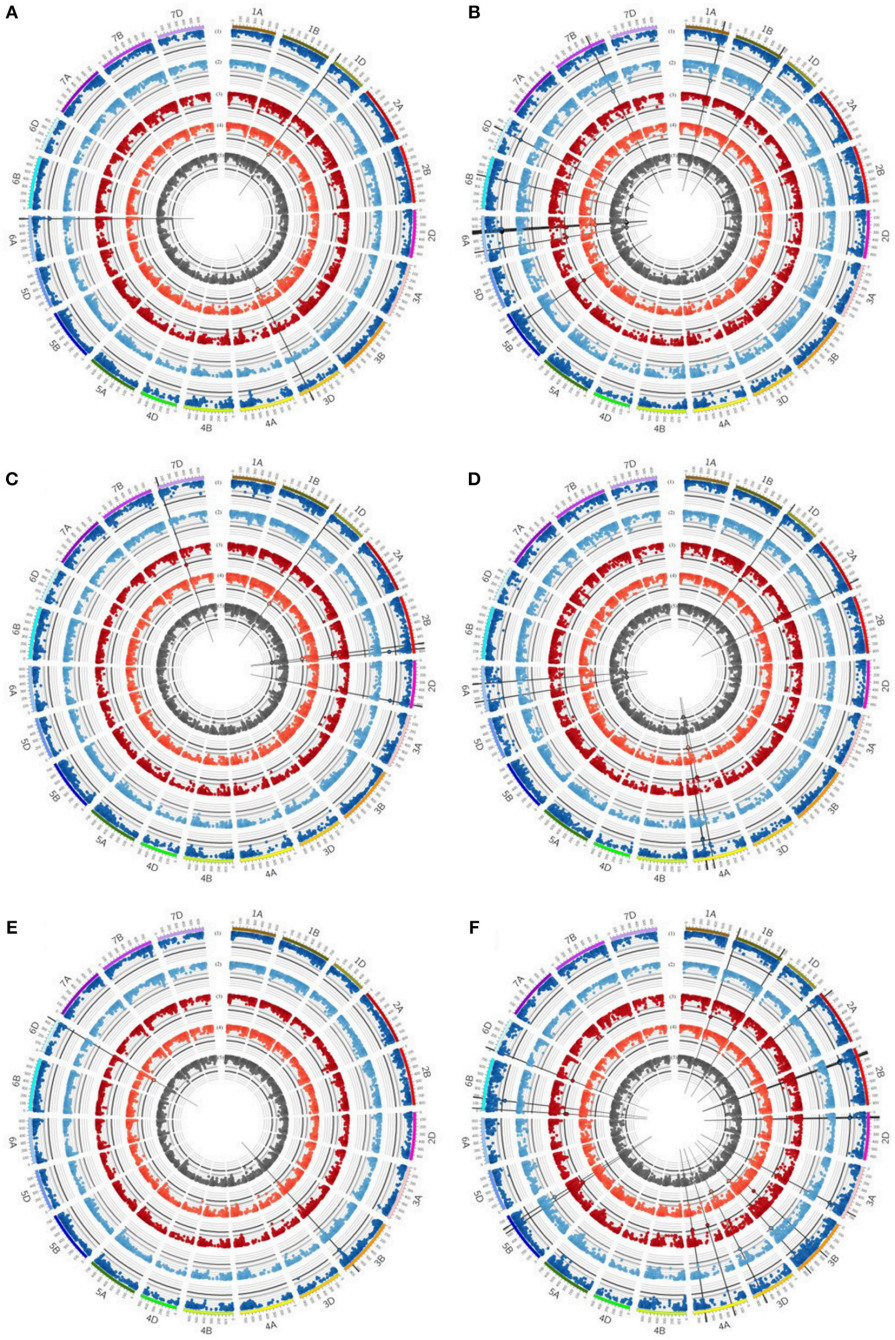
Circos plot for **(A)** grain yield (GY), **(B)** straw yield (SY), **(C)** number of grains per ear (GN), **(D)** biomass yield (BM), **(E)** thousand grain yield (TGW) and **(F)** harvest index (HI). Dark blue (1) and light blue (2) circles represent results of genome-wide association studies for traits under well watered conditions in the presence or absence of mycorrhizae, respectively. Dark red (3) and light red (4) circles represent results of genome-wide association studies for traits under drought stress conditions in the presence or absence of mycorrhizae, respectively. Dark gray (5) circle represents results of genome-wide association studies for traits across environments (combined). Genome-wide association study results of each trait pictured as Manhattan plot is based on 15511 polymorphic and mapped markers. Bold black line indicates threshold of significant marker trait associations with LOD 4.25. Significant marker trait associations are highlighted by black border. Putative quantitative trait locus regions are highlighted by vertical black lines.

Chromosomal regions on chromosomes 1A, 1B, 1D, 2A, 5B, 6A, 6B, 6D, and 7B are associated with SY. On chromosomes 1A and 7B, QTL regions were identified associated with SY under well watered conditions in the absence of mycorrhizae, whereas on chromosome 5B a QTL region was found associated with SY under drought stress conditions in the presence of mycorrhizae (Table 5, Figure 4, Supplementary Table 8). Six regions on chromosomes 6A, 6B, and 6D showed significantly associated MTAs for SY across all environments and under well watered conditions in the presence of mycorrhizae (Table 5, Figure 4, Supplementary Table 8). Another QTL on chromosome 2A is significantly associated with IT in the absence of mycorrhizae (Table 5, Supplementary Figure 6, Supplementary Table 8). On chromosomes 1B, 1D, and 6A four multi trait QTL regions were detected associated with SY as well as with BM or HI, respectively (Table 5).

For BM, significantly associated MTAs were identified on chromosomes 2A and 4A under drought stress conditions in the presence of mycorrhizae (Table 5, Figure 4, Supplementary Table 8). Additionally, on chromosome 4A another QTL was found to be associated with BM under drought stress conditions in the absence of mycorrhizae, BM under well watered conditions in the presence of mycorrhizae, STI in the presence of mycorrhizae and BM across all environments (Table 5, Figure 4, Supplementary Figure 6, Supplementary Table 8).

For EN, on chromosome 7D, one chromosomal region was identified, which is associated with drought stress related traits in general. This genomic region harbors significant MTAs for STI in the presence and absence of mycorrhizae (Table 5, Supplementary Figure 6, Supplementary Table 8).

QTL regions on chromosomes 3B and 6D are associated with TGW under well watered conditions in the presence of mycorrhizae as well as TGW under drought stress conditions in the absence of mycorrhizae, respectively (Table 5, Figure 4, Supplementary Table 8). Another QTL region associated with IT for TGW was located on chromosomes 7B (Table 5, Supplementary Figure 6, Supplementary Table 8).

**Figure 5:**
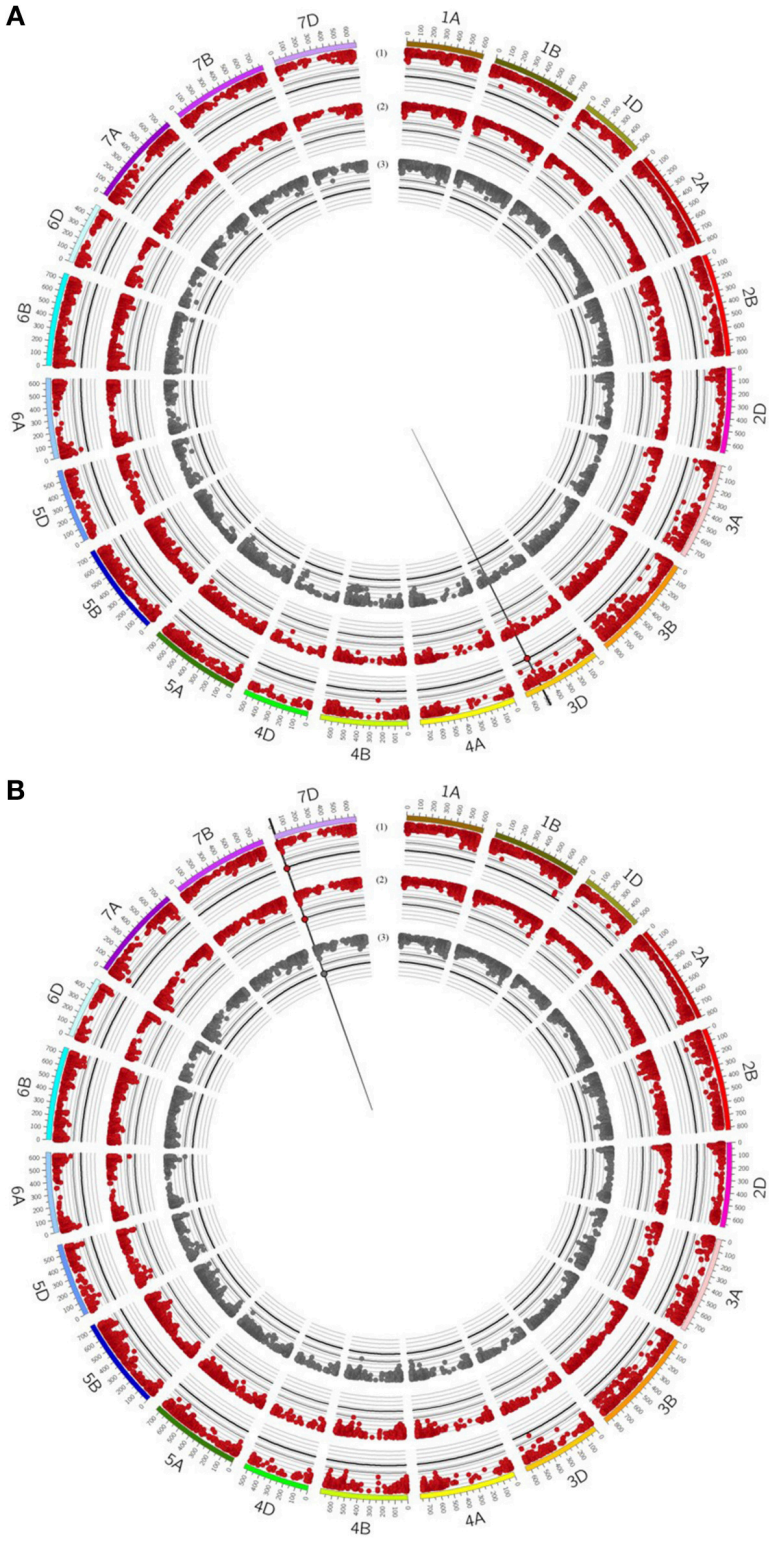
Circos plot for relative mycorrhizal responsivness (MR), absolute mycorrhizal responsivness (R) and deviations (δ) from the regression line of **(A)** grain yield (GY) and **(B)** number of grains per ear (GN). Dark red (1+2) circles represent results of genome-wide association studies for MR and R under drought stress condition. Gray (3) circle represents results of genome-wide association studies for δ under drought stress condition. Genome-wide association study results of each trait pictured as Manhattan plot is based on 15511 polymorphic and mapped markers. Bold black line indicates threshold of significant marker trait associations with LOD 4.25. Significant marker trait associations are highlighted by black border. Vertical black lines highlight putative quantitative trait locus regions.

For grain yield, significantly associated MTAs were detected on chromosomes 1D, 3D, and 6A (Table 5, Supplementary Table 8). On chromosome 6A, a chromosomal region was identified, which is associated with GY under well watered conditions as well as STI in the presence of mycorrhizae (Figure 4, Supplementary Figure 6). Furthermore, the QTL region on chromosome 1D is associated with GY under drought stress conditions in the absence of mycorrhizae, whereas the chromosomal region on chromosome 3D is associated with GY under drought stress in the absence of mycorrhizae, IT for GY in the absence of mycorrhizae as well as MR under drought stress conditions (Figure 4, Figure 5, Supplementary Figure 6). Additionally, both, QTL on chromosomes 1D and 3D are located in multi trait QTL regions also associated with GN under drought stress conditions in the absence of mycorrhizae and HI in the absence of mycorrhizae, respectively (Table 5, Supplementary Table 8).

Further QTL regions associated with HI were detected on several chromosomes. QTL regions on chromosomes 1B, 3B, 4A, 5B, 6B, and 6D are associated with HI under drought stress conditions in the presence of mycorrhizae, whereas QTL regions significantly associated with HI under well watered conditions in the presence of mycorrhizae were located on chromosomes 2A, 2B, 2D, 3A, and 6B (Table 5, Figure 4, Supplementary Table 8). Three other QTL on 3B, 3D, and 5B turned out to be significantly associated with HI under well watered conditions in the absence of mycorrhizae. The QTL on 3B and 3D are also found to be associated with HI under drought stress in the absence of mycorrhizae (Table 5, Figure 4, Supplementary Table 8).

For GN, a significantly associated chromosomal region on chromosome 2B was identified associated with GN across all environments and under drought stress (myco + and myco -) conditions (Figure 4, Table 5, Supplementary Table 8). In addition, significantly associated MTAs for STI (myco -) also located in this chromosomal region were detected (Supplementary Figure 6). Close to this QTL region, another QTL was found which is significantly associated with GN under well watered conditions in the presence of mycorrhizae (Figure 4, Table 5, Supplementary Table 8). Three additional QTL on chromosomes 1D, 2D, and 7D revealed associations with GN under drought stress conditions in the absence of mycorrhizae, GN under well watered conditions in the presence of mycorrhizae or under drought stress conditions in the presence of mycorrhizae, respectively (Figure 4, Table 5, Supplementary Table 8). Finally, a chromosomal region associated with response to mycorrhizae under drought stress conditions was identified on chromosome 7D (Figure 5, Table 5). This region is significantly associated with MR, R and δ.

**Figure 6:**
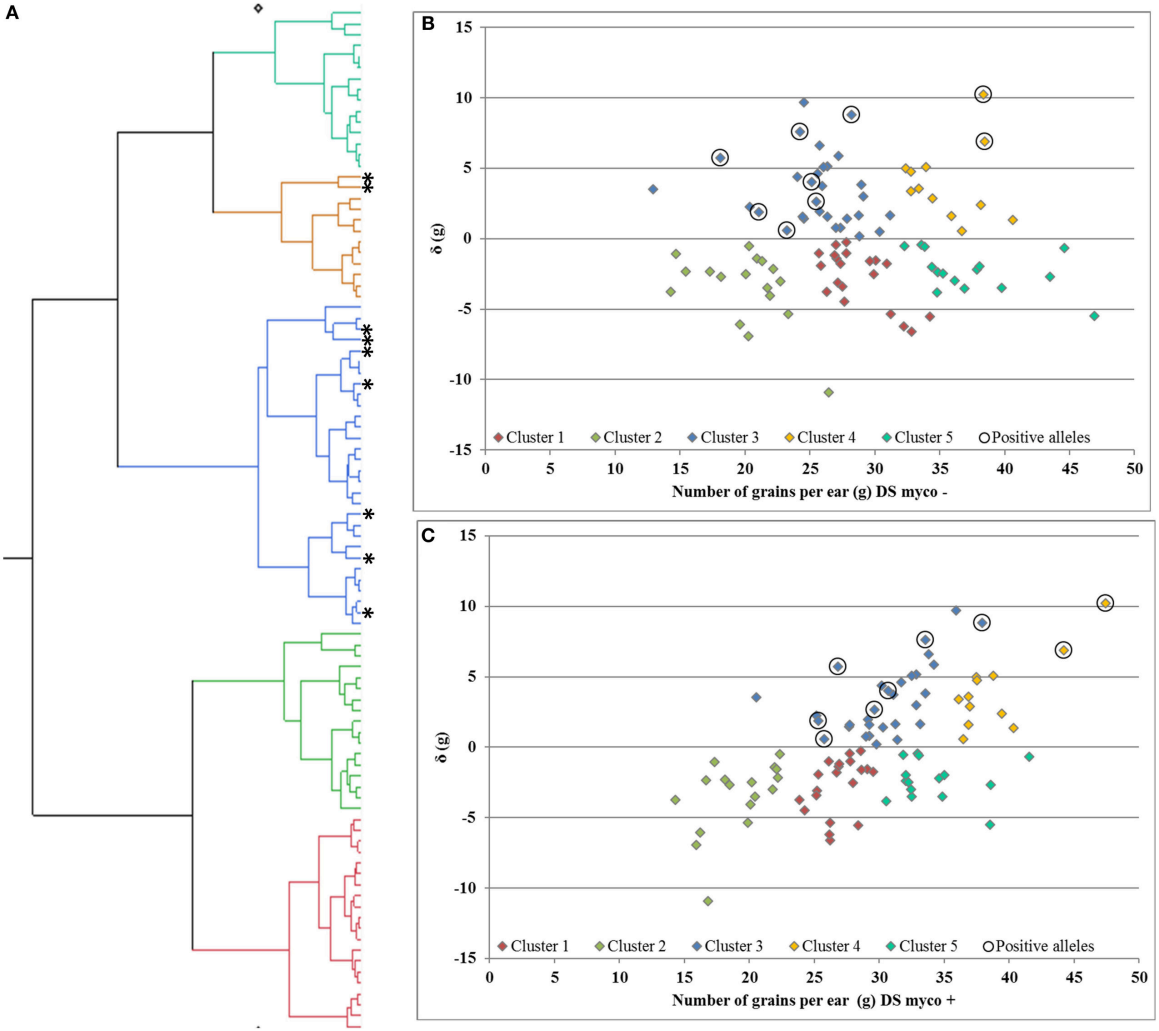
**(A)** Hierarchical cluster analysis and residual (δ) plots for regression of number of grains per ear under drought stress in the presence of mycorrhizae against number of grains per ear under drought stress in the **(B)** absence and **(C)** presence of mycorrhizae. Genotypes carries alleles positive associated with response to mycorrhizae highlighted by *and 0, respectively.

Deviation from the regression line is the most suitable measurement to identify genotypes with specific variation in response to mycorrhizae, as shown for GY (Supplementary Figures 2, 3). In total, only one QTL region on chromosome 7D (*QTL_*δ*_KN25_7D*) was identified, which is significantly associated with specific variation in response to mycorrhizae. In total, 10 % of genotypes carrying the positive allele (peak marker: RAC875_c19631_269; allelic effect: 5.92) showed an increased positive specific variation in response to mycorrhizae (Supplementary Figure 7). Hierarchical cluster analysis was implemented using genotype means of GN under drought stress conditions in the presence and absence of mycorrhizae as well as deviation from the regression line of GN under drought stress conditions in the presence of mycorrhizae against GN under drought stress conditions in the absence of mycorrhizae to identify clusters of genotypes associated with highly positive specific variation in response to mycorrhizae. Cluster analysis resulted in the detection of five clusters differing in GN and the specific variation in response to mycorrhizae (Figure 6). Cluster one, two and five consist of 20, 17 and 15 genotypes, which showed negative specific variation in response to mycorrhizae, whereas cluster three and four consist of 30 and 12 genotypes, which show positive specific variation in response of mycorrhizae. Interestingly, genotypes carrying the allele positively associated with an increased specific variation in response to mycorrhizae are all located in cluster three (7 genotypes) or four (two genotypes; Figure 6).

To summarize, the majority of significantly associated QTLs was detected only in one single environment, only five chromosomal regions were identified in two environments. QTL associated with drought stress tolerance-related traits mostly co-located with QTL of the underlying trait (i.e., QTL for drought stress related trait for GY is co-located with QTL for GY). Furthermore, only two QTL regions were identified associated with mycorrhizae responsiveness under drought stress conditions. These findings are not surprising considering the complex genetic basis of most traits under consideration and the complexity of interaction between wheat genotypes and mycorrhizal fungi under drought stress and well watered conditions. Furthermore, genes were identified to be located within the QTL regions detected under drought stress conditions based on the wheat reference genome of Chinese Spring (IWGSC, 2018). Thirteen (*QTL_RM25_3D-1*) to 305 (*QTL_GN25_1D and QTL_GY25_1D*) genes are located within respective QTL regions. Several of these genes are assumed to be associated with drought or osmotic stress according to the underlying GO terms (Supplementary Table 9). Additionally, transcription factor genes, which are known to be expressed in response to drought and genes coding for HSP or LEA proteins were detected in a number of QTL regions (Supplementary Table 9).

## Discussion

Since the 1980s, incidence of drought stress events increased significantly in the majority of wheat growing areas worldwide, caused by changes in trends in temperature and precipitation (Lobell et al., 2011; Coumou and Rahmstorf, 2012; Zampieri et al., 2017). Under drought stress conditions plant development and yield are negatively affected (reviewed in Farooq et al., 2014; reviewed in Osakabe et al., 2014; reviewed in Rao and Chaitanya, 2016). Therefore, further wheat breeding programs are faced with the challenge of producing new high yielding cultivars well adapted to climate changes in particular drought stress. Using new approaches e.g., the use of genotypic differences in response to mycorrhizae under drought stress conditions may be an option in this respect (Fester and Sawers, 2011; Galván et al., 2011). Detailed knowledge on the genetic basis of drought stress tolerance and the response to mycorrhizae may help to improve breeding for drought stress tolerance (Farooq et al., 2014; Wang and Qin, 2017). Therefore, this study focused on phenotypic and genetic differences of wheat in drought stress tolerance, and the response of wheat to mycorrhizae under drought stress conditions as well as the identification of QTL regions involved.

Negative effects of drought stress on wheat performance as well as genotypic differences in response to drought have been previously reported (e.g., Dencic et al., 2000; Dodig et al., 2012; Liu et al., 2017). Similar results were obtained for the set of genotypes investigated in this study, i.e., a significantly reduced performance under drought stress conditions but a broad variation concerning all traits analyzed. In particular, GY *per se* and GN were negatively affected under drought stress conditions, whereas TGW was significantly increased. It is known that terminal drought stress during flowering and grain filling is related to maximum yield losses (reviewed in Farooq et al., 2014), which is primarily caused by reduction in GN, rather than by a reduction of TGW (Dolferus et al., 2011; Dodig et al., 2012). These findings are in accordance with our results and have to be seen in the context of the negative impact of terminal drought stress on meiosis i.e., reduced fertility resulting in low grain set and therefore reduced GN (Onyemaobi et al., 2017). Additionally, it is to be assumed that the early induced terminal drought stress at booting stage indirectly increased TGW, as GN was significantly reduced and therefore available assimilates are partitioned to a low number of grains (Van Ginkel et al., 1998; Sanjari Pireivatlou and Yazdansepas, 2010). Other factors associated with the limitation of physiological and biochemical processes, i.e., premature leaf senescence, decrease in leaf water potential, stomatal closure, reduced net photosynthesis, oxidative damage of chloroplasts and reduced rates of carbon fixation and assimilate translocation are also involved in yield reduction under drought stress conditions (reviewed in Farooq et al., 2014; reviewed in Osakabe et al., 2014; reviewed in Rao and Chaitanya, 2016).

However, in this study it has been shown that inoculation of wheat with a mixture of mycorrhizal fungi significantly reduces the negative effects of drought stress. This positive effect was mainly caused by the substantial increase in GY and BM, whereby this generally positive effect of symbiosis was much weaker under well watered conditions. These findings are in accordance with previous reports, in which small sets of wheat genotypes were analyzed under field and green house conditions in this respect (e.g., Ellis et al., 1985; Al-Karaki et al., 2004; Moucheshi et al., 2012). Positive effects of mycorrhizal symbiosis on plant performance under drought stress conditions are assumed to be associated with reduced osmotic stress due to improved water and nutrient supply by the complex external fungal hyphae network in the context of changes in plant physiological and metabolic processes which are attributed to the fungal symbiont (Ruiz-Lozano et al., 2012; Li et al., 2014). Furthermore, improved root hydraulic properties, increased chlorophyll content as well as decrease in limitation of plant photosynthesis are reported for mycorrhizal plants due to an improved antioxidant activity, light absorbtion, and stomatal and mesophyll conductance (Ruiz-Lozano et al., 2012; Augé et al., 2015; Yooyongwech et al., 2016; Romero-Munar et al., 2017). However, these processes were not investigated here.

In recent years, several QTL regions on all wheat chromosomes were identified associated with physiological and agronomic traits under drought stress conditions (reviewed in Gupta et al., 2017). The majority of drought stress associated QTL regions were detected in bi-parental QTL mapping studies, which are limited by a low allelic diversity, a low recombination rate and the identification of QTL specific to the mapping population (Korte and Farlow, 2013; reviewed in Acuña-Galindo et al., 2015; reviewed in Gupta et al., 2017). However, in literature, some major and meta QTL regions as well as underlying candidate genes were reported assumed to be associated with drought stress tolerance of wheat (Acuña-Galindo et al., 2015; Gupta et al., 2017). Until now, these findings are rarely used in plant breeding (Gupta et al., 2017). In the present study, genomic regions significantly associated with GY and yield components as well as RM were identified under drought stress conditions in the presence and absence of mycorrhizae on several wheat chromosomes by GWAS. In the following section, these results will be discussed with results of previous studies. In this regard, the comparison of QTL regions identified by different marker systems on different genetic maps is difficult (Edae et al., 2014; Chen et al., 2016). Therefore, comparisons between studies were conducted based on chromosomes. Additionally, to improve the compatibility between the genetic and physical map, QTL regions identified by SNP markers with known flanking sequences were remapped to the reference genome of Chinese Spring (IWGSC, 2018). QTL regions associated with root dry mass under drought stress conditions in the presence of mycorrhizae were detected on chromosomes 3A, 3B, and 3D. Liu et al. (2013) reported also QTL for several seedlings root traits on 3A and 3B under drought stress conditions. A multi trait QTL region was identified associated with BM in the presence of mycorrhizae and drought stress index based on SY in the presence of mycorrhizae on chromosome 1D. Additionally, two genome regions were detected on chromosome 2A and 4A associated with BM in the presence of mycorrhizae. Acuña-Galindo et al. (2015) also found meta QTL under drought stress conditions on chromosomes 1D, 2A, and 4A associated with BM and other traits. For TGW under drought stress conditions, one QTL region was detected associated with TGW in the absence of mycorrhizae on chromosome 6D. This is in accordance with findings of Lopes et al. (2013) who reported a significant marker trait association for TGW under heat stress and well watered conditions on chromosome 6D. QTL regions associated with HI in the presence or absence of mycorrhizae were detected on chromosomes 1B, 3B, 3D, 4A, 5B, 6B, and 6D. In literature, several QTL regions on different chromosomes were reported for HI under drought stress conditions (e.g., Hill et al., 2013; Acuña-Galindo et al., 2015; Ain et al., 2015; Shukla et al., 2015; Xu et al., 2017), which are in line with the present findings. Two multi trait QTL regions were found on 1D and 3D in the absence of mycorrhizae, associated with GY and GN or GY and the HI, respectively. Several authors also found QTL or meta QTL regions on these chromosomes associated with GY under drought stress conditions (Bennett et al., 2012; Zorić et al., 2012; Acuña-Galindo et al., 2015; Shukla et al., 2015). Furthermore, Shukla et al. (2015) also reported a multi trait QTL region associated with HI and GY on chromosome 3D.

To summarize, comparison of chromosomal location of QTL regions identified under drought stress conditions in this study with those reported in previous studies revealed that some of the identified QTL are located on chromosomes, previously described to be associated with the trait of interest under drought stress conditions. This confirms the reliability of the present findings, but also new QTL regions for drought stress tolerance were detected. Moreover, most of the significantly associated QTLs for GY and yield components were found only in a single environment and seem to be environment specific, therefore, whereby QTL associated with drought stress tolerance related traits mostly co-located with QTL of the underlying trait. It is not surprising that traits under extremely different environmental conditions linked to different QTL considering the complex genetic basis of these traits and the complexity of interaction between wheat genotypes and mycorrhizal fungi under drought stress and well watered conditions (Kaeppler et al., 2000; Maccaferri et al., 2011; Pinto and Reynolds, 2015).

The effect of arbuscular mycorrhizal fungi on plant performance depends on the balance between costs and benefits. Therefore, interaction between fungus and plant is considered as a continuum between parasitism and mutualism, depending on environmental conditions, the plant species and genotype as well as the interaction between plant genotype and mycorrhizae species (Johnson et al., 1997, 2015). Differences in the effect of mycorrhizal symbiosis on plant performance associated with environments differing in nutrient availability is well documented (Kaeppler et al., 2000; Mohammad et al., 2004; Shukla et al., 2012) and genotypic differences in the response to mycorrhizae were reported for several plant species including wheat (Azcon and Ocampo, 1981; Manske, 1990; Hetrick et al., 1992; reviewed in Tawaraya, 2003; Yücel et al., 2009). Early studies already suggested that the ability to form mycorrhizal symbiosis and the response of plants to mycorrhizae varied between genotypes and that these are both heritable traits under polygenic control (Manske, 1990; Mercy et al., 1990). It was also stated, that the level of root colonization and plant response to mycorrhizae were not correlated (Manske, 1990; Kapulnik and Kushnir, 1991). This is consistent with results of this study. Under drought stress and well watered conditions, a broad genotypic variation in the response to mycorrhizae as well as in root colonization of wheat by mycorrhizal fungi was observed, but no strong positive correlation between both traits. Interestingly, the positive effect of symbiosis on plant performance increased under drought stress conditions compared to well watered conditions, even though root colonization of wheat by mycorrhizal fungi was reduced under drought stress conditions. Consequently, there is some evidence, that both traits are under control of different genomic regions and that the effective use of symbiosis depends on a balanced interaction between plant and fungi as well as environmental conditions rather than the level of root colonization by mycorrhizal fungi.

QTL studies in maize and *Allium* revealed genome regions associated with response to mycorrhizae (Kaeppler et al., 2000; Galván et al., 2011). To the best of our knowledge, until now, no genomic regions associated with response to mycorrhizae were identified in wheat by QTL studies or GWAS.

In literature several terms and underlying equations exist in order to evaluate the effect of mycorrhizae on plant performance (Janos, 2007). In the present study relative mycorrhizal responsiveness (Hetrick et al., 1992) and absolute mycorrhizal responsiveness (Janos, 2007; Sawers et al., 2010) were calculated for each genotype to evaluate the effect of mycorhhizal symbiosis on yield and yield components of wheat. Two QTL regions on chromosomes 3D and 7D were associated with relative mycorrhizal responsiveness based on GY and GN. Hetrick et al. (1996) and Yücel et al. (2009) also used relative mycorrhizal responsiveness to evaluate the effect of mycorrhizal fungi on biomass production of substitution lines of hexaploid and tetraploid wheat, involving individual chromosomes of mycorrhizal responsive hexaploid and tetraploid wheat donors, respectively. These studies gave first hints about chromosomal location of mycorrhizal responsiveness genes in wheat, but findings are contradictory. In wheat, Hetrick et al. (1995) reported that the chromosomes 1A, 5B, 6B, 7B, and 7D of the mycorrhizal responsive donor cultivar Cheyenne caused improved response to mycorrhizal symbiosis in the background of the mycorrhizal non-responsive cultivar Chinese Spring. Furthermore, it is assumed that major genes associated with mycorrhizal responsiveness are located on 5B and 7B (Hetrick et al., 1995). In the present study, the three marker trait associations with the lowest *P*-values for relative mycorrhizal responsiveness based on BM were located on 1A and 6B. In contrast, Yücel et al. (2009) found that chromosomes of the B genome of the wild emmer wheat donor (*Triticum turgidum* subsp. *dicoccoides*) have a greater negative impact on mycorrhizal responsiveness in the background of the durum wheat (*Triticum turgidum* L. var. *durum*) cultivar Langdon than chromosomes of the A genome.

In accordance with Sawers et al. (2010) and Galván et al. (2011), it has been shown that relative or absolute mycorrhizal responsiveness are not suitable as breeding objectives to improve the positive interaction between wheat and mycorrhizal fungi, as this will result in the selection of plants showing reduced performance in the absence of mycorrhizae. Following the assumptions of Sawers et al. (2010), variation in response to mycorrhizae was partitioned in a common and a specific component by using a linear regression model. Specific variation in response to mycorrhizae was defined as deviations (δ) from the regression of the performance of genotypes under drought stress conditions in the presence of mycorrhizae against performance of genotypes under drought stress conditions in the absence of mycorrhizae (Sawers et al., 2010). The specific variation in response to mycorrhizae appears to be a more suitable breeding objective, because this trait can be used to select promising genotypes and improve the interaction between wheat and mycorrhizae as well as plant performance under drought stress conditions without the reduction of plant performance in the absence of mycorrhizae (Sawers et al., 2008, 2010). It is suggested that genomic regions associated with a specific variation in response to mycorrhizae will allow the identification of mycorrhizae responsive genotypes carrying alleles of genes associated with a specific response to mycorrhizae that may be used in plant breeding approaches. In this context, only one QTL region associated with the specific variation in response to mycorrhizae based on GN was identified on chromosome 7D. Interestingly, this QTL region is also associated with relative and absolute mycorrhizal responsiveness based on GN. In total, 270 genes are located in this QTL region. However, it is not possible to deduce which one is the functional gene associated with the specific response to mycorrhizae. This is due to the fact that the significantly associated peak marker (RAC875_c19631_269) may be located either directly within the respective candidate gene or is in LD with the causal locus (Rafalski, 2010; Bush and Moore, 2012). Therefore, further investigations are needed. Today, the availability of the wheat reference genome of Chinese Spring (IWGSC, 2018) opens new possibilities, e.g., the identification of differentially expressed genes located in the QTL region by combining a QTL approach with whole-genome microarray expression analysis as shown for poplar (*Populus* ssp.; Labbé et al., 2011). Moreover, recently published proteome and transcriptome studies in wheat and maize gave first hints to the modulation of the bread wheat and durum root proteome under drought stress conditions in the presence or absence of mycorrhizae and on the regulation of gene expression of aquaporines under drought stress conditions in the presence and absence of mycorrhizae, respectively (Bernardo et al., 2017; Quiroga et al., 2017). As a prospect for further research, Bernardo et al. (2017) detected 50 proteins differentially expressed in the bread wheat cultivar Chinese Spring under drought stress conditions in the presence and absence of mycorrhizae. Interestingly, one of these significantly down regulated proteins (sucrose:fructan 6-fructosyltransferase) found under drought stress conditions in the presence of mycorrhizae is located within the QTL region *QTL_*δ*_GN25_7D* which is significantly associated with the specific response to mycorrhizae. Sucrose:fructan 6-fructosyltransferase is associated with osmoprotection of membranes under drought stress conditions, as it is a key enzyme in fructane biosynthesis (Livingston et al., 2009; Bernardo et al., 2017). It is assumed that down regulation of this protein under drought stress conditions in the presence of mycorrhizae is associated with increased drought stress tolerance of plants due to mycorrhizal root colonization (Bernardo et al., 2017). The QTL peak marker RAC875_c19631_269 is located 2Mbp downstream of the gene coding for sucrose:fructan 6-fructosyltransferase. It is now appropriate to examine the interrelation between these findings.

## Conclusions

It has been shown that the inoculation of wheat with mycorrhizal fungi significantly improves drought stress tolerance and that genotypic differences in the specific response of plants to mycorrhizae under drought stress conditions exist. QTL regions were identified associated with grain yield and yield components as well as drought stress tolerance-associated traits in the presence and absence of mycorrhizae. Additionally, one QTL region was detected associated with the specific response of wheat plants to mycorrhizae under drought stress conditions, which is assumed to be used in applied wheat breeding. In general, it can be expected that only a subset of major QTL regions associated with the traits of interest was identified. This is because of the suggested highly quantitative nature of traits under investigation influenced by several small effect QTLs in combination with the reduced power of GWAS due to the observed moderate heritability of some investigated traits as well as the low number of genotypes under investigation. Further research is necessary to validate detected QTL regions and associated candidate genes. However, this study represents the starting point of the discovery of candidate genes associated with drought stress tolerance and the specific response to mycorrhizae under drought stress conditions as well as the development of useful gene-based functional markers for wheat breeding to speed up the improvement of drought stress tolerant wheat cultivars.

## Author Contributions

AS, WF, and FO planed and designed the research. HL performed experiments and all other analyses. HL and FO wrote the manuscript. HL, AS, WF, and FO contributed to the interpretation of results and read and approved the final manuscript.

### Conflict of Interest Statement

The authors declare that the research was conducted in the absence of any commercial or financial relationships that could be construed as a potential conflict of interest.

## Abbreviations

ANOVA: analysis of variance
AP2/ERF: APETALA2/Ethylene response element binding factor family
AREB/ABF: abscisic acid-responsive element binding proteins family
BM: aboveground biomass yield per plant
bZIP: basic leucine zipper
CMLMK: Compressed mixed linear model corrected for K matrix
DSI: drought stress susceptibility indices
EN: number of ears per plant
GN: Number of grains per ear
GO: Gene ontology
GWAS: genome-wide association studies
GY: grain yield per plant
h^2^: Broad sense heritability
HI: Harvest index (HI)
HSP: heat shock proteins
IT: tolerance index
K matrix: kinship matrix
LD: linkage disequilibrium
LEA: late embryogenesis abundant
LOESS: smooth locally weighted polynomial regression
Lsmeans: least square means
MAF: minor allele frequency
MR: relative mycorrhizal responsiveness
MSD: mean squared difference
MTAs: marker trait associations
myco +: presence of mycorrhizae
myco −: absence of mycorrhizae
MWC: maximal soil water capacity
MYB: myeloblastosis
MYC: myelocytomatosis
PCoA: principal coordinate analysis
PIC: polymorphism information content
QTL: quantitative trait loci
R: absolute mycorrhizal responsiveness
r^2^: squared allele frequency correlation
RD: Rogers' distances
RM: root dry mass per pot
rp: phenotypic correlation coefficient (Pearson)
SNP: single nucleotide polymorphism
STI: stress tolerance index
SY: straw yield per plant
TGW: Thousand grain weight
δ: Deviations from the regression line.
