# Supplementary material for: Genome-Wide Association Studies Reveal Genomic Regions Associated With the Response of Wheat (Triticum aestivum L.) to Mycorrhizae Under Drought Stress Conditions
Source: Front Plant Sci. 2018 Dec 4;9:1728. doi: 10.3389/fpls.2018.01728 (PMC6290350; doi:10.3389/fpls.2018.01728)
Supplement: Supplementary file 1 [file Image_1.pdf]

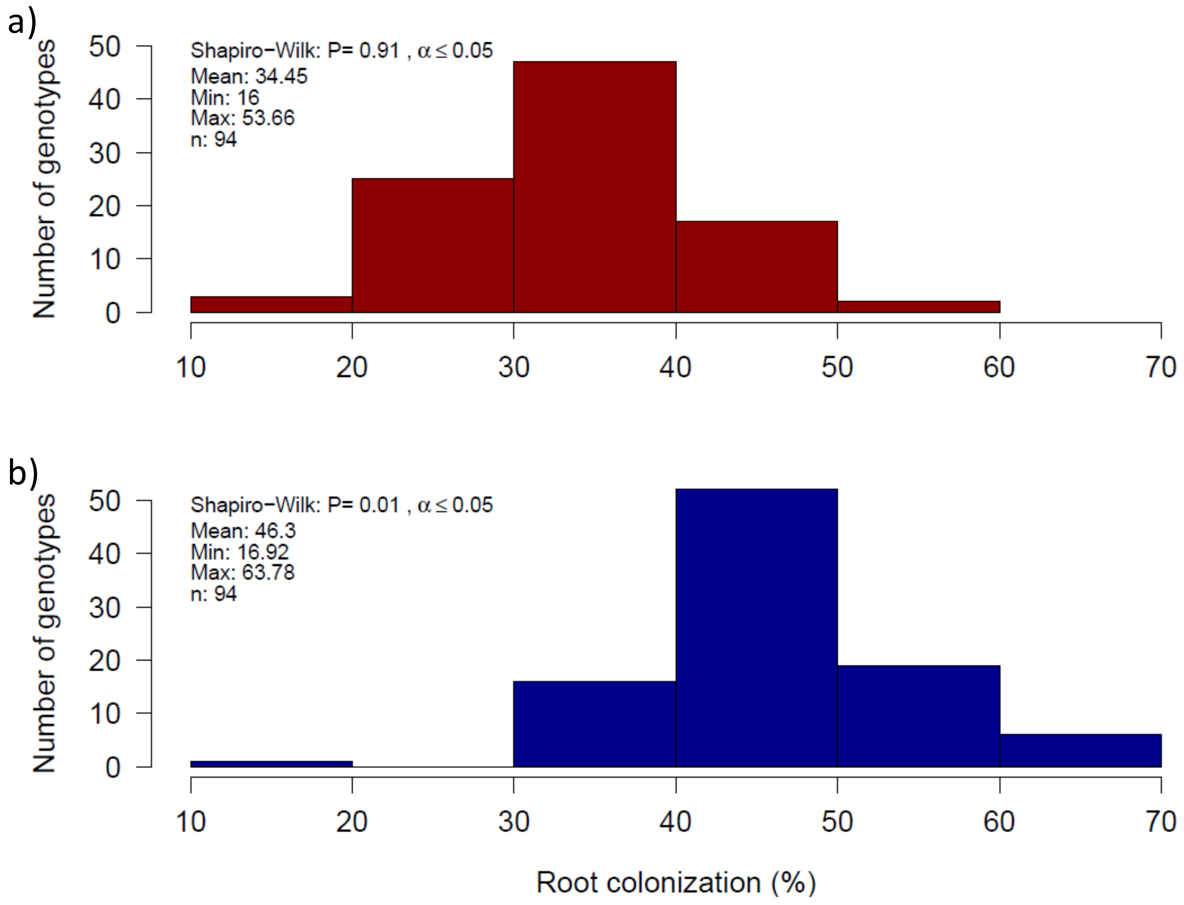

Suppl. figure 1: Mean, minimum (Min), maximum (Max) and distribution of genotype means for root colonization a) under drought stress and b) well watered conditions by mycorrhizal fungi for n = 94 wheat genotypes evaluated in two years.
