# Supplementary material for: Genome-Wide Association Studies Reveal Genomic Regions Associated With the Response of Wheat (Triticum aestivum L.) to Mycorrhizae Under Drought Stress Conditions
Source: Front Plant Sci. 2018 Dec 4;9:1728. doi: 10.3389/fpls.2018.01728 (PMC6290350; doi:10.3389/fpls.2018.01728)
Supplement: Supplementary file 2 [file Image_2.pdf]

a)

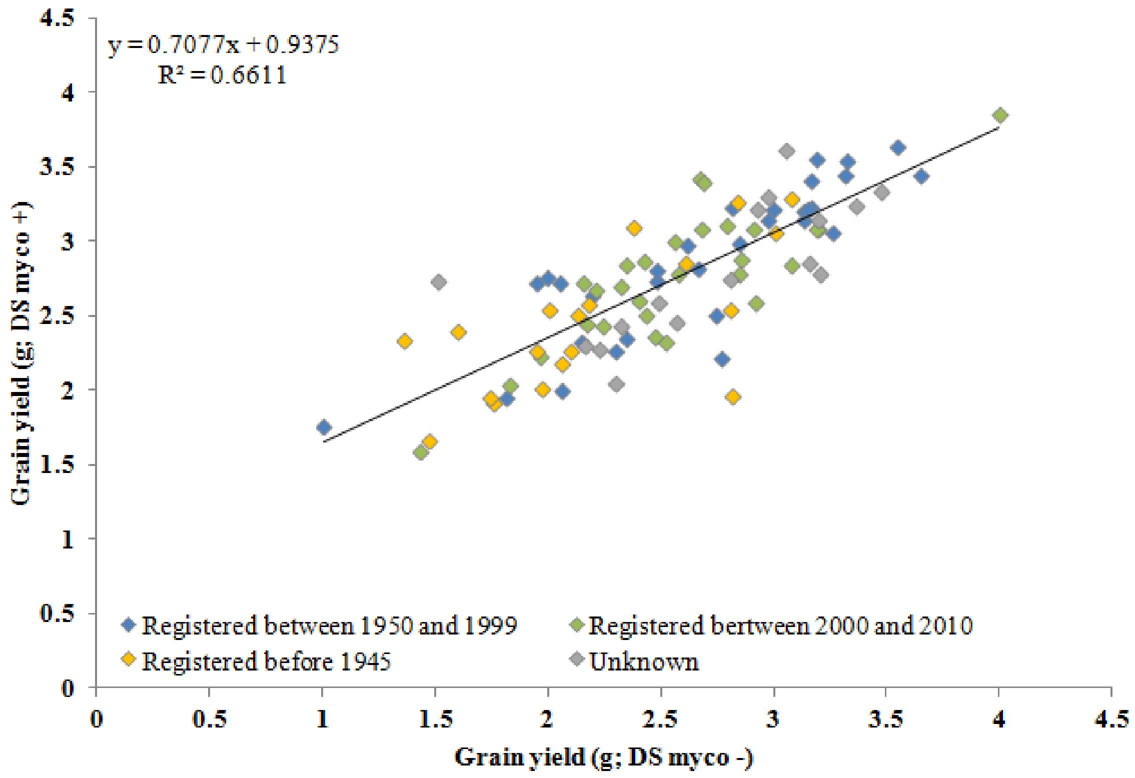
y = 0.7077x + 0.9375
R² = 0.6611
Grain yield (g; DS myco +)
Grain yield (g; DS myco -)
Registered between 1950 and 1999
Registered bertween 2000 and 2010
Registered before 1945
Unknown
0
0.5
1
1.5
2
2.5
3
3.5
4
4.5

b)

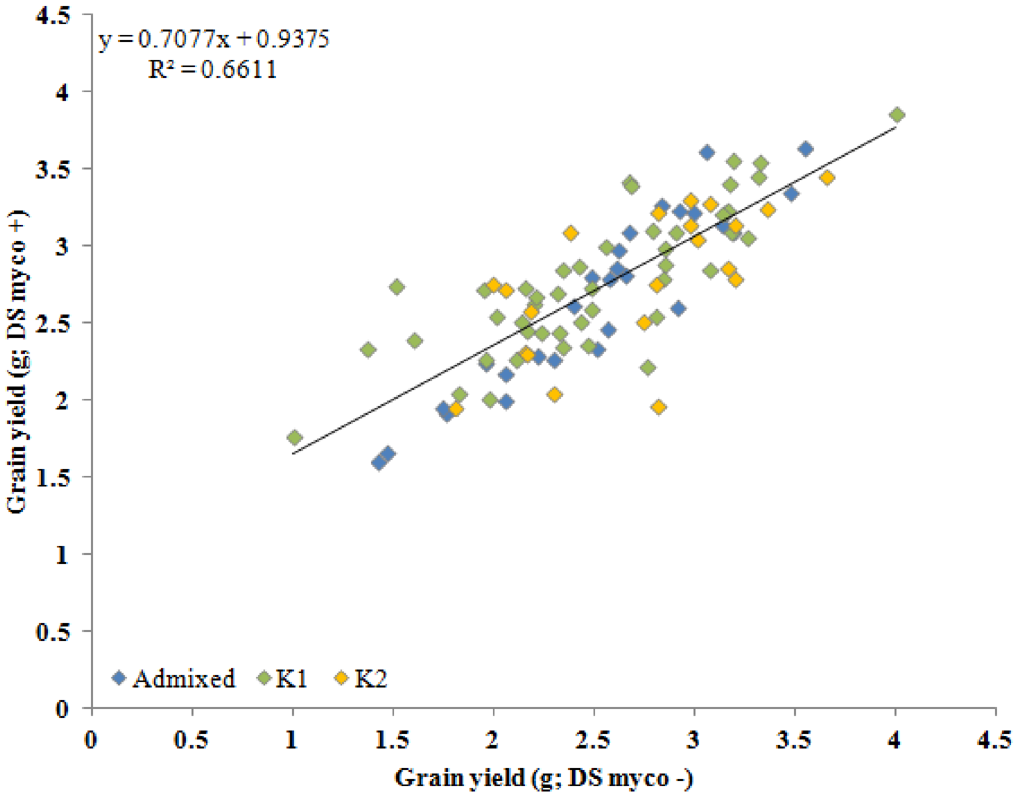
y = 0.7077x + 0.9375
R² = 0.6611
Grain yield (g; DS myco +)
Grain yield (g; DS myco -)
Admixed
K1
K2
0
0.5
1
1.5
2
2.5
3
3.5
4
4.5

Suppl. figure 2: Linear regression of grain yield (GY) under drought stress conditions in the presence of mycorrhizae (DS myco +) against grain yield (GY) under drought stress conditions in the absence of mycorrhizae (DS myco -) for the 94 genotypes. Genotypes were assigned to groups according to the a) year of release and b) structure grouping. K1: Structure group 1, K2: Structure group 2 (For details please see Lehnert *et al*. 2017).
