# Supplementary material for: Genome-Wide Association Studies Reveal Genomic Regions Associated With the Response of Wheat (Triticum aestivum L.) to Mycorrhizae Under Drought Stress Conditions
Source: Front Plant Sci. 2018 Dec 4;9:1728. doi: 10.3389/fpls.2018.01728 (PMC6290350; doi:10.3389/fpls.2018.01728)
Supplement: Supplementary file 3 [file Image_3.pdf]

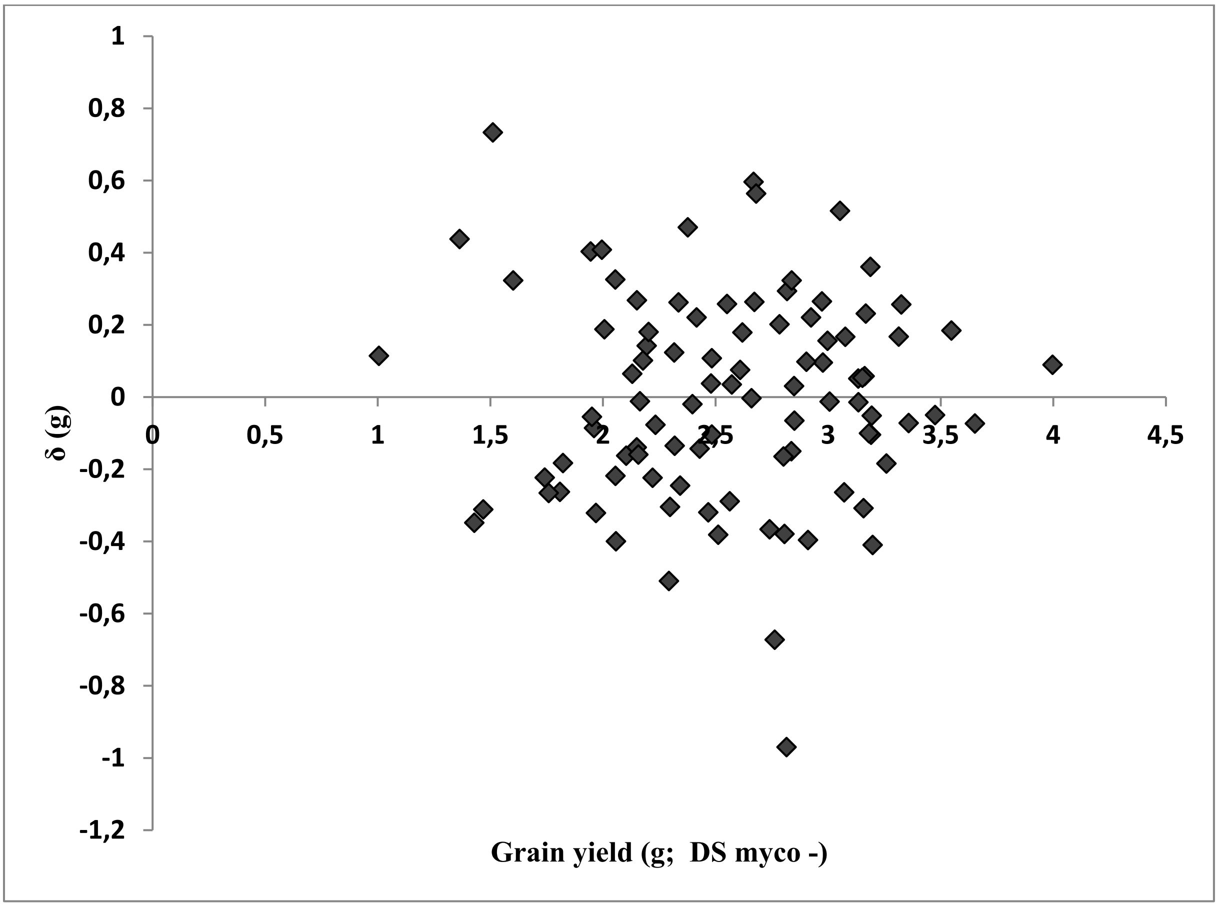

Suppl. figure 3: Residual (δ) plot of regression of grain yield (GY) under drought stress conditions in the presence of mycorrhizae against grain yield (GY) under drought stress conditions in the absence of mycorrhizae (DS myco -) for 94 wheat genotypes.
