# Supplementary material for: Genome-Wide Association Studies Reveal Genomic Regions Associated With the Response of Wheat (Triticum aestivum L.) to Mycorrhizae Under Drought Stress Conditions
Source: Front Plant Sci. 2018 Dec 4;9:1728. doi: 10.3389/fpls.2018.01728 (PMC6290350; doi:10.3389/fpls.2018.01728)
Supplement: Supplementary file 4 [file Image_4.pdf]

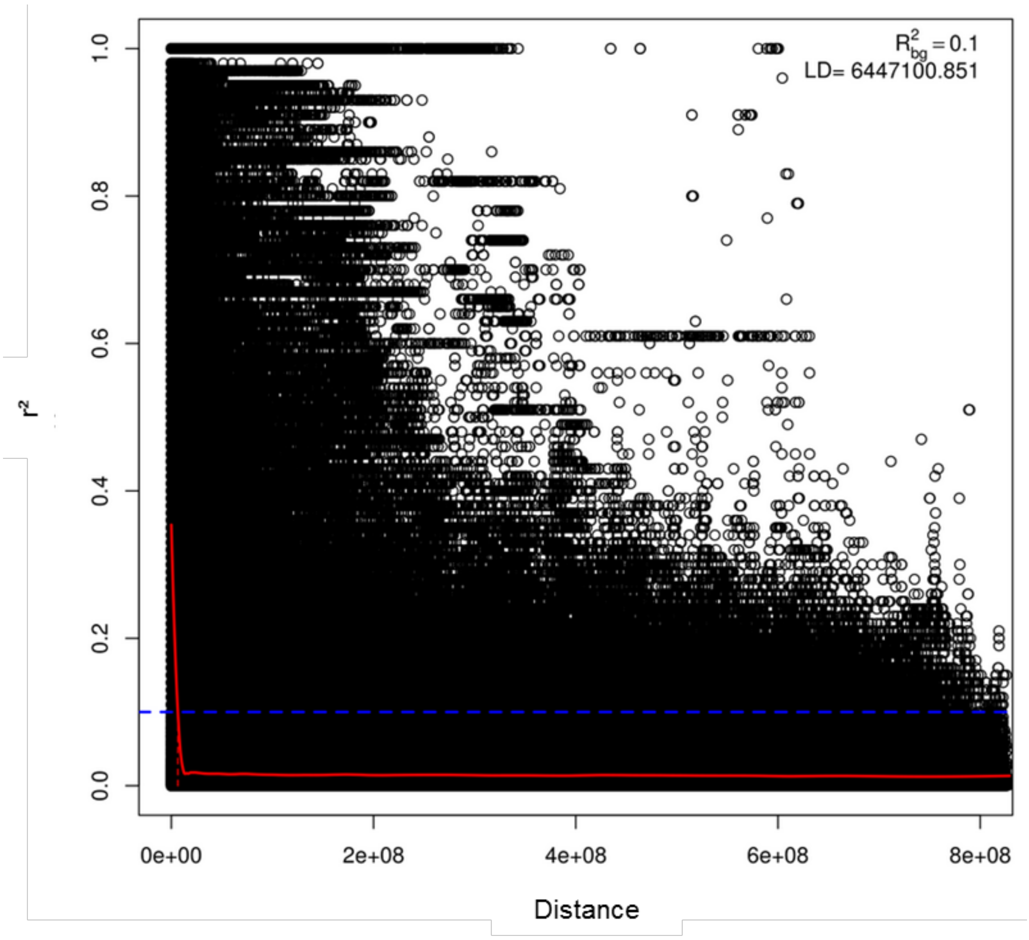

Suppl. figure 4: Whole genome linkage disequilibrium (LD) decay plot showing the distribution of pairwise squared allele frequency correlation (r^2) values plotted against the distance in base pairs (Distance) of SNP marker loci in 94 winter wheat genotypes. For LD-decay all marker-marker combinations within one chromosome where considered and the distance for each combination was calculated with regard to the mapping position of each marker. The critical r^2 value ($R^2_{bg} = 0.1$) is indicated by a dashed blue line and the smooth locally weighted polynomial regression (LOESS) curve by a solid red line. LD decay is the intersection point of the LOESS curve with the critical r^2 value resulting in 6,447,100bp.
