# Supplementary material for: Genome-Wide Association Studies Reveal Genomic Regions Associated With the Response of Wheat (Triticum aestivum L.) to Mycorrhizae Under Drought Stress Conditions
Source: Front Plant Sci. 2018 Dec 4;9:1728. doi: 10.3389/fpls.2018.01728 (PMC6290350; doi:10.3389/fpls.2018.01728)
Supplement: Supplementary file 5 [file Image_5.pdf]

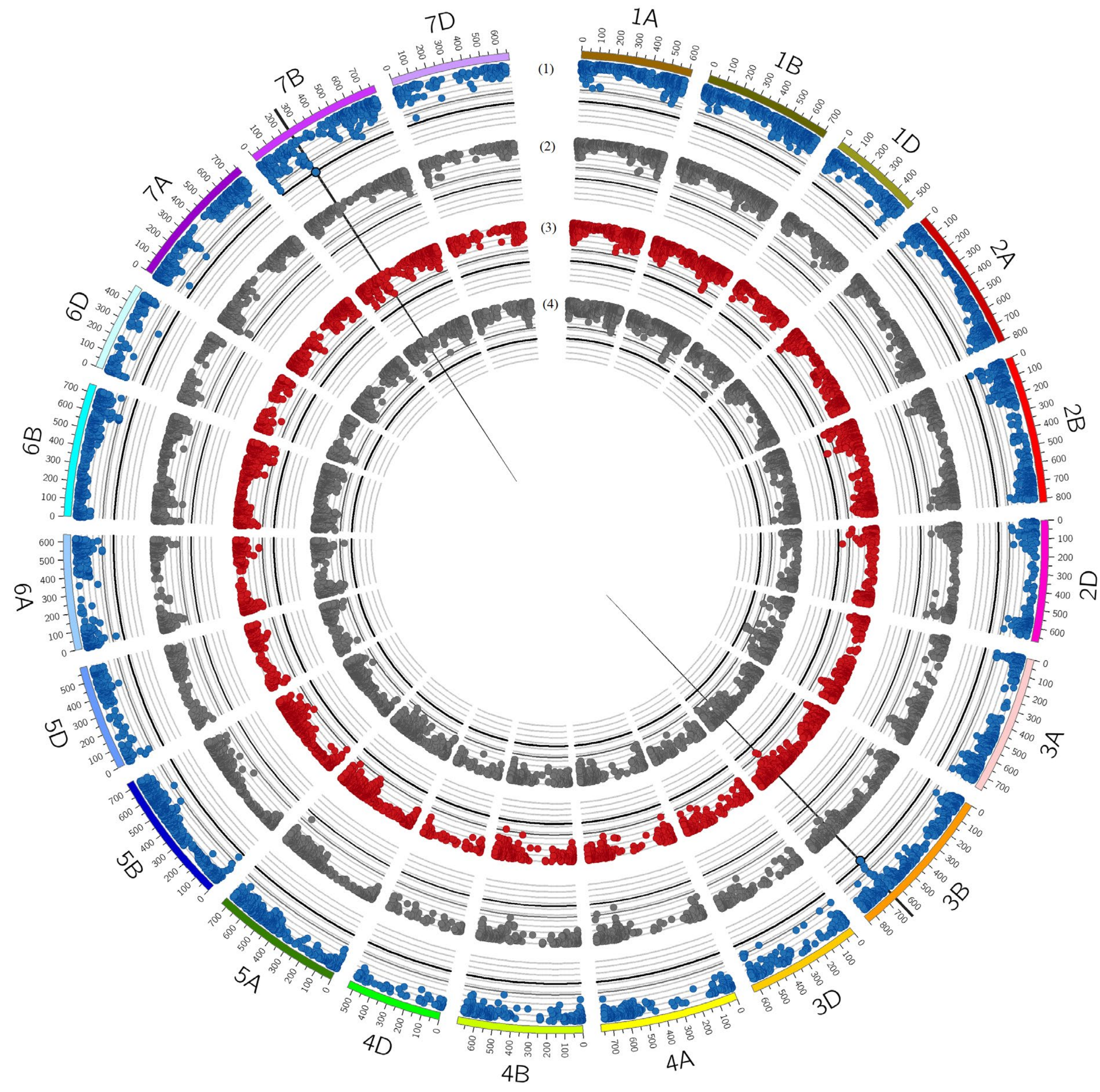

Suppl. figure 5: Circos plot for root colonization and root dry mass (RM). Dark blue (1) and dark (3) red circles represent results of genome-wide association studies for root dry mass under drought stress or well watered conditions in the presence of mycorrhizae. Grey (2+4) circles represent results of genome-wide association studies for root colonization under well watered and drought stress conditions. Genome-wide association study results of each trait are shown as Manhattan plot based on 15511 polymorphic and mapped markers. Bold black line indicates threshold of significant marker trait associations with LOD 4.25. Significant marker trait associations are highlighted with a black border. Vertical black lines highlight putative quantitative trait locus regions.
