# Supplementary material for: Genome-Wide Association Studies Reveal Genomic Regions Associated With the Response of Wheat (Triticum aestivum L.) to Mycorrhizae Under Drought Stress Conditions
Source: Front Plant Sci. 2018 Dec 4;9:1728. doi: 10.3389/fpls.2018.01728 (PMC6290350; doi:10.3389/fpls.2018.01728)
Supplement: Supplementary file 6 [file Image_6.pdf]

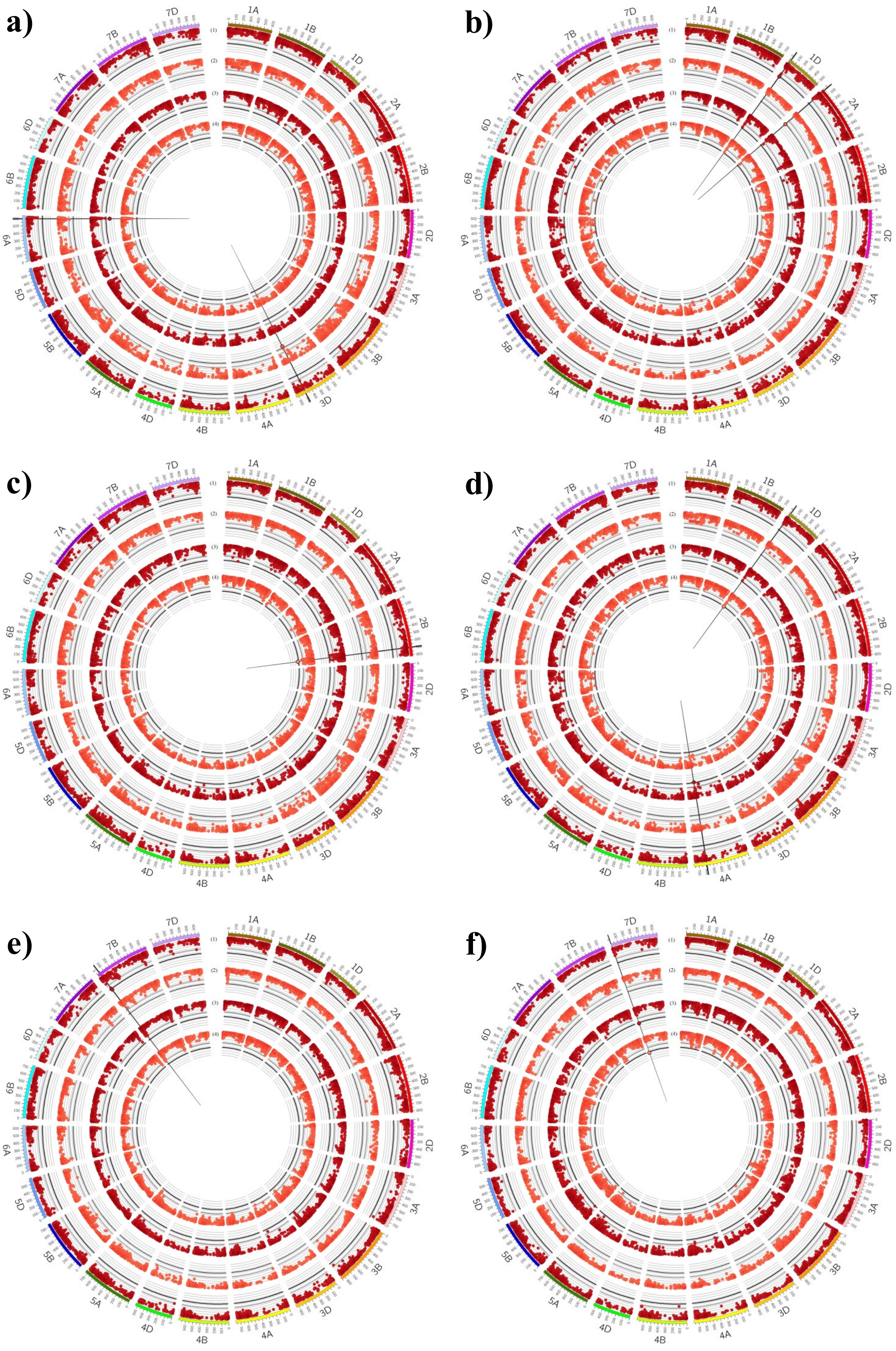
a)
b)
c)
d)
e)
f)
(1)
(2)
(3)
(4)
1A
1B
1D
2A
2B
2D
3A
3B
3D
4A
4B
4D
5A
5B
5D
6A
6B
6D
7A
7B
7D

Suppl. figure 6: Circos plot for tolerance index (IT) and stress tolerance index (STI) of a) grain yield (GY), b) straw yield (SY), c) number of grains per ear (GN), d) biomass yield (BM), e) thousand grain yield (TGW) and f) number of ears per plant (EN). Dark red (1) and light red (2) circles represent results of genome-wide association studies for tolerance index (IT) in the presence or absence of mycorrhizae, respectively. Dark red (3) and light red (4) circles represent results of genome-wide association studies for stress tolerance index (STI) in the presence or absence of mycorrhizae, respectively. Genome-wide association study results of each trait are shown as Manhattan plot based on 15511 polymorphic and mapped markers. Bold black line indicates threshold of significant marker trait associations with LOD 4.25. Significant marker trait associations are highlighted with a black border. Vertical black lines highlight putative quantitative trait locus regions.
