# Supplementary material for: Genome-Wide Association Studies Reveal Genomic Regions Associated With the Response of Wheat (Triticum aestivum L.) to Mycorrhizae Under Drought Stress Conditions
Source: Front Plant Sci. 2018 Dec 4;9:1728. doi: 10.3389/fpls.2018.01728 (PMC6290350; doi:10.3389/fpls.2018.01728)
Supplement: Supplementary file 7 [file Image_7.pdf]

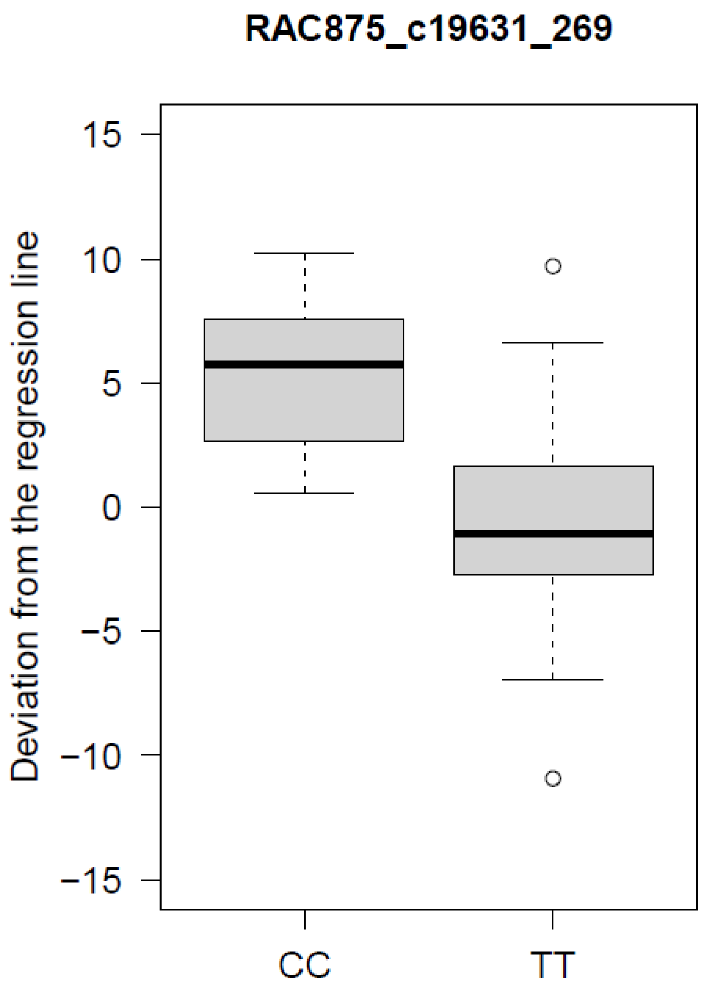

Suppl. figure 7: Boxplots of genotype means for the deviation from the regression line based on number of kernels per ear for genotypes with the CC or TT allele at the QTL marker RAC875_c19631_269.
